# Evaluation of a targeted anti-α_v_β_3_ integrin near-infrared fluorescent dye for fluorescence-guided resection of naturally occurring soft tissue sarcomas in dogs

**DOI:** 10.1007/s00259-024-06953-x

**Published:** 2024-10-22

**Authors:** Patricia Beer, Paula Grest, Christiane Krudewig, Chris Staudinger, Stefanie Ohlerth, Carla Rohrer Bley, Armin Jarosch, Houria Ech-Cherif, Enni Markkanen, Brian Park, Mirja Christine Nolff

**Affiliations:** 1 Clinic for Small Animal Surgery, Vetsuisse Faculty, University Animal Hospital, University of Zurich, Zurich, Switzerland; 2 Institute of Veterinary Pathology, Vetsuisse Faculty, University of Zurich, Zurich, Switzerland; 3 Clinic for Diagnostic Imaging, University Animal Hospital, Vetsuisse Faculty Zurich, University Zurich, Zurich, Switzerland; 4 Division of Radiation Oncology, University Animal Hospital, Vetsuisse Faculty Zurich, University Zurich, Zurich, Switzerland; 5 Department of Pathology, Charité-Universitätsmedizin Berlin, Corporate Member of Freie Universität Berlin and Humboldt-Universität zu Berlin, Berlin, Germany; 6 Institute of Veterinary Pharmacology and Toxicology, Vetsuisse Faculty, University of Zurich, Zurich, Switzerland

**Keywords:** Second window sarcoma imaging, Targeted near-infrared fluorescent imaging, Dog, Targeted near-infrared fluorescent dye, Comparative oncology, Soft tissue sarcoma

## Abstract

**Purpose:**

Complete resection is a key prognostic factor for survival in patients with soft tissue sarcoma (STS), in humas and companion animals alike. Fluorescence-guided surgery could improve resection accuracy. As dogs are frequently affected by STS, they serve as a model to test an anti-α_v_β_3_ integrin targeting near-infrared fluorescent (NIRF) dye (Angiostamp^TM^800) for fluorescence-guided surgery in STS to evaluate its safety and feasibility in dogs, and if it translates into a clinically relevant benefit compared to the standard of care with regards to completeness of surgery and local recurrence. Furthermore, we aimed to correlate target expression and NIRF-signal intensity.

**Methods:**

Twenty dogs with STS were randomly allocated to either receive Angiostamp™ (NIRF group) or physiologic saline (control group) preoperatively. The researchers were blinded for treatment, and resections were adapted based on the NIRF-signal, if needed. Margin status was histologically determined at the 1 and 3 cm margin. The tumor-to-background ratio was measured in native tissue biopsies and formalin-fixed tissue. The fluorescent area was compared to the corresponding tumor areas as confirmed by histology using the Dice coefficient. Target expression was quantified by immunohistochemistry and correlated to NIRF-signal ratios.

**Results:**

A fluorescent signal was detected in all 10 tumors of the NIRF group, with a tumor-to-background ratio of 7.4 ± 5.8 in native biopsies and 13.5 ± 10.9 in formalin-fixed tissue. In the NIRF group, resection margins were adapted in 5/10 cases, leading to complete resection and preventing R1 in four of these cases. In the NIRF and control group 9/10 and 8/10 resections were R0, with one local recurrence in each group and one sarcoma-related death in the NIRF group. The NIRF-signal correlated with the histologically confirmed tumor area (Dice coefficient 0.75 ± 0.17). Target expression was higher in tumor compared to peritumoral tissue (*p* < 0.0003) and showed a moderate correlation with the NIRF-signal (*r* = 0.6516, *p* < 0.0001).

**Conclusion:**

Fluorescence-guided surgery using Angiostamp™ can pinpoint residual disease in the tumor bed and contributes to an improved resection accuracy in canine STS.

**Supplementary Information:**

The online version contains supplementary material available at 10.1007/s00259-024-06953-x.

## Introduction

Soft tissue sarcomas (STS) are frequent mesenchymal malignancies in dogs representing 10%-15% of diagnosed skin and subcutaneous tumors [1]. Importantly, STS in dogs share a high level of molecular similarity with their human counterparts and therefore represent highly accessible models for clinical translation [2]. Surgical resection is the standard treatment for localized STS with the aim of complete tumor removal (R0 resection) in both dogs and humans [3–9]. The optimal surgical dose is under debate [4, 5], but a lateral 2–3 cm margin and one fascial plane beneath the tumor or a radical resection is mostly recommended for dogs [10, 11].

Unfortunately, the surgeon’s ability to precisely delineate the tumor during surgery is limited, as locoregional invasion and formation of microscopic lesions in the surrounding healthy tissue cannot be visualized, leading to incomplete resection in up to 50% of cases [3, 4]. This issue must be addressed, as R0 resection is the only prognostic factor a surgeon can directly influence. Infiltrated margins are a significant predictor of local recurrence and strongly associated with survival [4, 5]. In dogs local recurrence rates after complete resections are around 9.8%, clean but close resections show 22.5% local recurrence and incomplete resections result in local recurrence rates of 33.3% [3, 12]. A highly accurate imaging tool, designed for intraoperative tumor margin evaluation, that enables the surgeon to visualize the tumor in real time could be a game changer. Optical imaging during surgery using near-infrared fluorescence (NIRF) could offer real-time high-resolution guidance [13, 14]. In this context, tumor-selective probes recognizing tumor specific molecular signatures are of great interest [13].

Angiostamp™ is a NIRF dye that targets α_v_β_3_ integrin, a transmembrane cell adhesion receptor. α_v_β_3_ integrin is overexpressed in activated angiogenic endothelial cells during tumor neoangiogenesis and in certain tumor cells. It is known to be a driver of tumor progression, tumor cell proliferation and metastasis [15–17], and has already been used as a target for imaging and treatment of solid tumors [18]. Angiostamp™'s RGD (arginine-glycine-aspartic acid) peptide moieties show a high affinity and selective integrin receptor binding resulting in a good tumor-to-normal tissue contrast in preclinical trails [17, 19–21]. A first investigation using this probe in 12 feline fibrosarcoma patients documented reliable accumulation resulting in R0 resections in all cats [17]. Unfortunately, no control group was included, and evaluation of dye performance was limited to tumor-to-background ratio (TBR) and resection status. The aim of this randomized controlled blinded clinical study was to validate if fluorescence-guided surgery using Angiostamp™ is safe and feasible in dogs, and if it translates into a clinically relevant benefit compared to the standard of care, regarding completeness of surgery and local recurrence. Furthermore, we aimed to correlate target expression and NIRF-signal intensity.

## Materials and Methods

### Patient selection and treatment allocation

The study was carried out in accordance with the Swiss Animal Welfare legislation and approved under the license number ZH198/2020. Client owned dogs presented for surgical resection of a histologically or cytologically confirmed primary or recurrent STS were included if complete resection was deemed feasible and if owner consent was available. Distant metastasis, local extension precluding R0 resection, as well as presence of kidney disease were exclusion criteria. Routine staging was performed including contrast-enhanced computed tomography (CT) of the tumor and thorax in 19/20 dogs. One dog with peripheral nerve sheath tumor underwent magnetic resonance imaging (MRI). CT imaging features and tumor volume were evaluated using an ellipsoid formula with the linear diameters of the tumor and in addition volumetrically with the open-source software application “3D Slicer” (version 5.4.0) [22] (Supplementary Information (SI) 1). Patients were randomly allocated to the treatment group using the method of minimization, to balance potential confounding variables (tumor localization, tumor size, sex, body weight). The investigators and owners were blinded to treatment allocation until completion of data analysis.

**Fig. 1:**
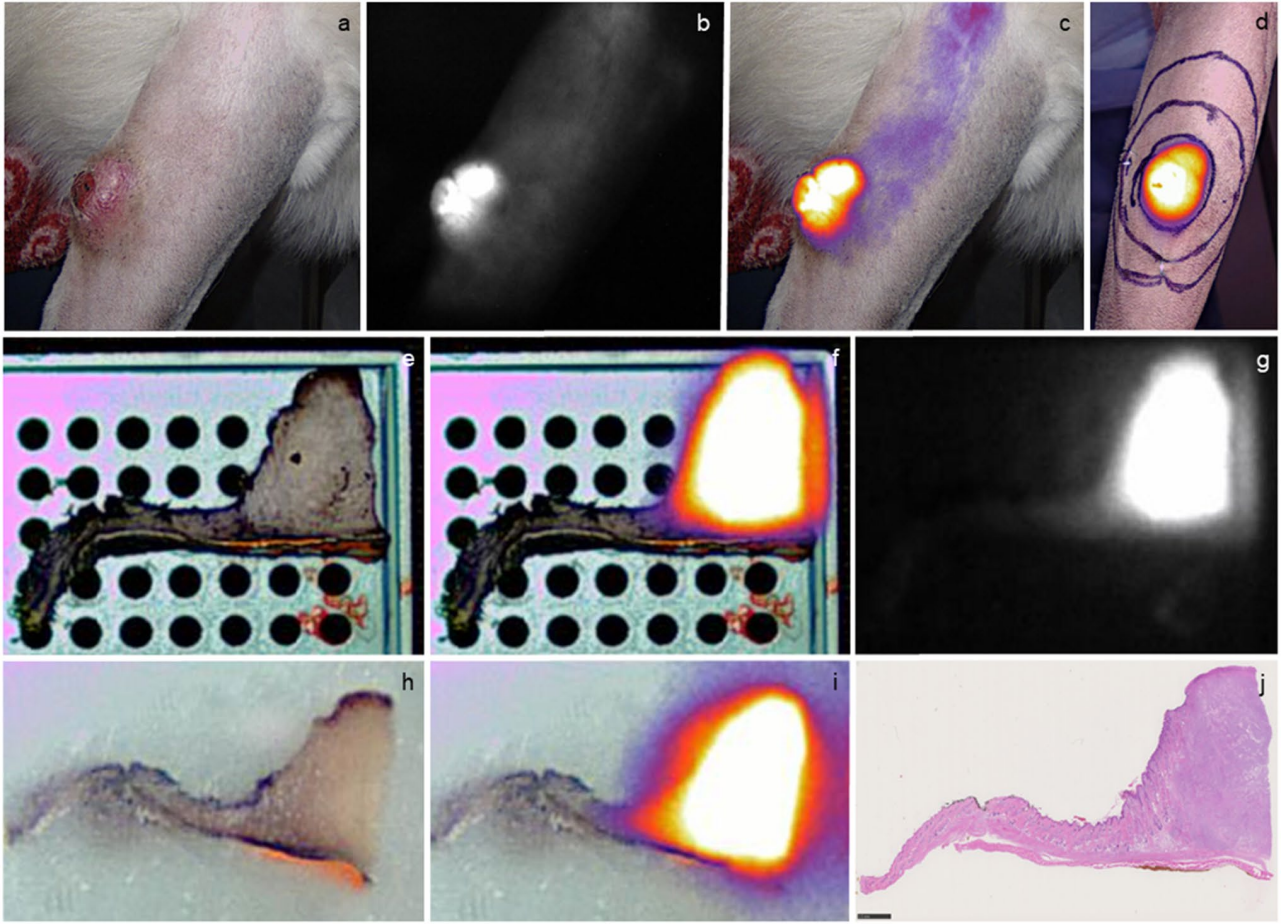
(**a-c**) Preoperative NIRF imaging of a superficial STS on the front limb of a dog with unpigmented skin. The white light image (**a**), the IR image (**b**) and color fused image **(c)** show an immediate fluorescent dye uptake after injection of Angiostamp™. (**d**) Intraoperatively prior to the tumor resection the tumor, and the 1 cm and 3 cm margin around the tumor were encircled on the skin. (**e–g**) The formalin-fixed tissue and the corresponding paraffin-embedded tissue (**h, i**) was fluorescent in the area of the tumor as confirmed by histology (**j**)

Dogs in the NIRF group received an intravenous injection of Angiostamp™ (0.15 mg/kg) (Fluoptics, Grenoble, France), while the control group received sterile saline of an equivalent volume (0.15 ml/kg) as sham treatment. Dogs were continuously monitored until surgery for the occurrence of side effects including measurement of the heart frequency, respiratory rate, blood pressure, and body temperature, auscultation of heart and lungs, evaluation of mucous membranes (color, capillary refilling time), local or generalized swelling and oedema, occurrence of nausea, vomiting or other unexpected events. Furthermore, transcutaneous tumor imaging was performed using an open-field fluorescence imaging device VisionSense™ VS3 Iridium System (Medtronic, Minneapolis, USA). It provides a white light image (Fig. 1a), a grey scale NIRF image (IR image) (Fig. 1b) and pseudo-colored overlay image (color fused image) (Fig. 1c).

### Surgical procedure and postoperative care

Wide local excision (3 cm lateral margins and one fascial plane) was planned in cases with tumors originating in the subcutis, while compartmental resection was performed in cases with large tumors of the extremities. All surgeries were performed by one board-certified veterinary surgeon (M.N.) in the time period from July 2021 to May 2023. The 3 cm resection margin was marked around the suspected tumor dimension on the skin (Fig. 1d). In cases with detectable NIRF-signal, the margin was adjusted to include a minimum margin of 3 cm around all fluorescent tissue. In addition, a 1 cm margin around the suspected tumor (control group) or NIRF-signal (NIRF group) was marked. Tumor resection was completed under fluorescent control to include clearly fluorescent tumor tissue. After resection, the specimen was reimaged at a standardized distance of 30 cm to visualize the superficial and deep margin. NIRF imaging of the would bed was undertaken to identify residual fluorescent tissue. If a positive NIRF signal was detected within the tumor bed, these fluorescent tissue areas were either completely resected or biopsies were taken in case vital structures (e.g. neuro-vascular bundles) were affected or if resection would have added a major morbidity to the patient (e.g. functional impairment, change of the would closure technique).

Clinical follow-up was performed at day 10 and every three months in the first year after surgery, every six months in the second year and once a year thereafter. A control CT scan was recommended six months after surgery.

### Confirmation of molecular targeting

**Fig. 2:**
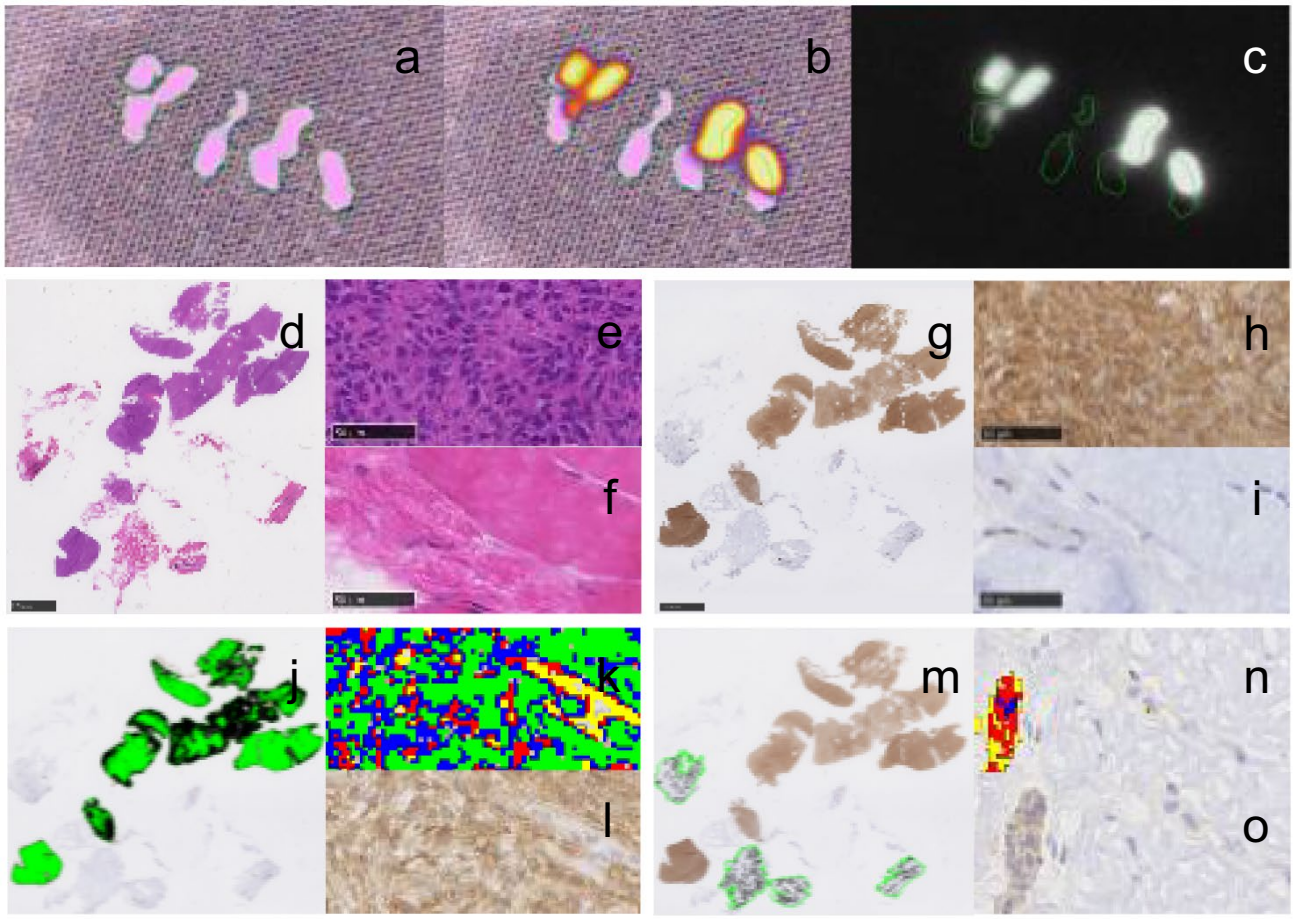
**a-c** NIRF imaging of native tissue biopsies was performed. The white light image was used to determine the ROI of visible tissue (green line) (**a**) which was then transferred to the color fused (**b**) and IR image for measurement of the fluorescence intensity (**c**). Tumor (**e**) was highly fluorescent (**b, c**) while the peritumoral tissue (**f**) was non-fluorescent as confirmed by histology (**d-f**). Visual assessment (**g-i**) confirmed overexpression of α_v_β_3_ integrin in tumor (**h**) compared to peritumoral tissue (**i**). Histomorphometry was used to delineate regions of interest (ROI). Tumor (**j-l**) and peritumoral tissue (**m–o**) were separately delineated in two different ROIs. The HE stained sections (**d-f**) served as control for tissue differentiation. The area of anti-α_v_β_3_ integrin labelled tissue and the overall mean staining intensity (MI) within the ROIs was then calculated (**h-n**)

One to three biopsies were taken from the tumor, the 1 cm margin, the 3 cm margin, and the tumor bed. All biopsies were imaged on a black table (working distance of 30 cm (Fig. 2a-c)). After NIRF imaging, tissue biopsies were cryoembedded and hematoxylin and eosin (HE) staining (Fig. 2d-f) and immunohistochemistry (IHC) were performed to assess α_v_β_3_ integrin expression (Fig. 2 g-o; SI2).

The primary anti-integrin α_v_β_3_ antibody, clone LM609 (MAB1976; RRID:AB 2925190) used for IHC was validated for dogs by Reverse Transcription quantitative PCR (RT-qPCR) and immunofluorescence staining using canine sarcoma cell lines (SI3).

#### Analysis of α_v_β_3_ integrin expression

Qualitative and semi-quantitative evaluation of α_v_β_3_ integrin expression was performed by a board-certified veterinary pathologist (P.G.) and a trained veterinarian and PhD student (P.B.) applying previously described criteria [23]. A final expression score ranging from no expression to low, intermediate, and high expression was determined. Quantitative image analysis was performed using the automated histomorphometry software Visiopharm® (Hoersholm, Denmark) [23]. A detailed description of the image analysis steps is available in Fig. 2, and SI2 [23]. A target selection criteria score was applied to assess if α_v_β_3_ integrin resembles a suitable target for fluorescence-guided surgery (SI2 Table 1) [23, 24].

### Assessment of NIRF accuracy

#### Diagnosis and grading

The tumors were trimmed using a modified bread loafing technique (SI4 Fig. 7). In fluorescent tumors, trimming was performed under NIRF imaging control. Formalin-fixed tissue (FFT) sections were imaged on a black table prior to paraffin embedding. Routine diagnostics was performed by P.G.. Further IHC analyses were done, if required for STS subtype identification. Surgical margins were assessed for their completeness using the residual disease (R) classification system [25] with R0 resections defined as microscopically complete tumor resections, R1 as microscopically incomplete with neoplastic cells reaching the resection margin, and R2 as macroscopically incomplete resections. In addition, the distance between tumor and the surgical margin was measured in mm. For tumors of the NIRF group, an additional STS subtype classification was completed by a trained human pathologist (A.J.).

#### Subjective correlation of NIRF-signal and true tumor extent

NIRF images of the FFT sections of the trimmed tumor specimen were used to subjectively assess the correspondence of a positive NIRF-signal with histologically confirmed tumor tissue (Fig. 2e-j). P.G. and P.B. reviewed all histological slides for the presence (neoplastic) or absence (non-neoplastic) of tumor tissue. The corresponding IR images of the FFT slices were visually reviewed by P.B. for the presence (positive) or absence (negative) of a visible fluorescent signal (SI4).

#### Calculation of signal-to-background ratios in native tissue

**Fig. 3:**
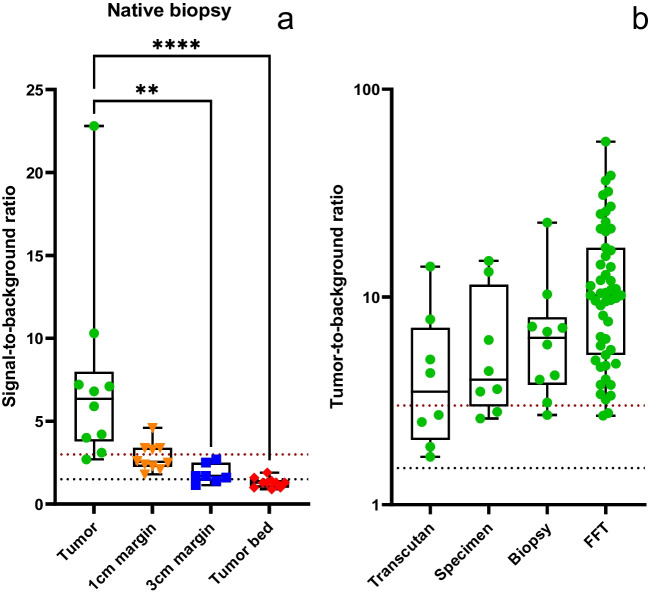
Boxplot showing the SBR in native tissue biopsies of tumor, 1 cm, and 3 cm margin and tumor bed (**a**). Boxplot (**b**) shows the TBR of the tumor specimens at the various stages of processing (transcutaneous imaging, imaging after resection, native biopsy, and after formalin fixation). The dotted black line delineates a ratio of 1.5, the red delineates a ratio of 3 [26]

Ratios of the mean fluorescent signal intensity (MFI) in defined ROIs were calculated to compare the MFI between tissue types. Signal-to-background ratios (SBR) were defined as the MFI ratio between the tumor ROI and the MFI of the set ROIs for different margins, background and tumor bed. The MFI was determined in the IR image (72 dpi, 24 bits per pixel (RGB)) and displayed as mean, minimum, maximum and standard deviation measured in arbitrary units (Pixel) ranging from 0 to 256. The TBR was calculated by comparing the MFI of the tumor ROI to a ROI of unaffected background representing either peritumoral tissue or an area without tissue on the black table. TBR was determined for native tumor biopsies and native tumor tissue before and after resection. A ratio of ≥ 3 was considered sufficient for clear delineation of tumor, ratios between 3 and 1.5 were considered suboptimal, and a ratio below 1.5 insufficient [26]. A detailed description of NIRF image analysis is provided in SI4. ROI registration and MFI measurements were performed using ImageJ 1.53 k (National Institutes of Health, Bethesda, MD, USA; RRID:SCR_003070). In white light images of the native tissue biopsies a ROI was delineated to include all visible tissue of each biopsy site (tumor, peritumoral 1 and 3 cm margin, background), the ROI was transferred to the IR image and a MFI ratio for each comparison (tumor vs. 1 cm, tumor vs. 3 cm, and tumor versus background without tissue) was calculated (SI4 Fig. 3). To determine the ratios during surgery, images taken before and after tumor resection were used to set ROIs in the tumor, a distant area of unaffected skin (background), and the tumor-bed after resection. In the latter, areas of high NIRF-signal were encircled in addition. The ROIs were then superimposed to the corresponding NIRF images and MFI was determined for each ROI. The same process was repeated on images of the excised specimen (skin side up) with the background set in a region without tissue. Ratios were calculated for tumor vs. background (SI4 Fig. 2–6).

#### Calculation of signal-to-background ratios in FFT and correlation of NIRF-signal and tumor extent

SBR calculation for FFT sections was done using images taken after sectioning of the tumor specimen directly prior to embedding in paraffin. As paraffin-embedding causes a halo effect, determination of NIRF-signal in paraffin embedded tissue was not possible. Detection of fluorescence intensity in microscopic sections after mounting was not successful. Therefore, the most accurate comparison of fluorescent area and histologically confirmed tumor or non-affected tissue (detected in HE stained sections) necessitated the comparison of NIRF-signal intensity in the FFT section prior to paraffin embedding, that corresponds to the histologic slide used for delineation of tumor margins. This required correction of artefacts in sample geometry that had occurred during mounting. To correct for distorted tissue, a custom-made program that allowed shape matching of the tissue outline in the FFT image and the histologic section was created using MATLAB R2023a (Mathworks Natick, Massachusetts, USA; RRID:SCR_001622) (SI4 Fig. 8). Only samples that included a tumor to normal interface were included in this analysis. After shape matching, the following areas were compared between the histologic slide and the IR image of the corresponding FFT section: histologically confirmed tumor, histologically confirmed non affected peritumoral tissue, and background ROI. The latter was set in an area where no tissue was visible (SI4 Fig. 9).

The tumor-to-peritumoral tissue ratio was then calculated. To compare the correspondence between areas with NIRF-signal and tumor, the Dice coefficient was used as similarity measurement (SI4 Fig. 10). The Dice coefficient of the tissue ROIs, HE tissue and all visible tissue, was calculated to assess the accuracy of the shape matching.

### Statistical analysis

Quantitative data were expressed in means ± SD. As data was not normally distributed, a nonparametric test was used for unpaired samples (Mann–Whitney for two groups or Kruskal–Wallis-test for more than 2 groups) to compare means of the signal ratios and of integrin-stained areas and MI pixel values. Following a Kruskal–Wallis-test, Dunn’s post-hoc analysis was carried out on each pair of groups to adjust the p-value for multiple testing. Data are presented as boxplots with the centerline indicating the median, the whiskers depicting the minimum and maximum values and the bounds of the box define the 25th to 75th percentiles. Correlation between the IHC expression of α_v_β_3_ integrin and the SBR of the native tissue biopsies was assessed by the Spearman’s coefficient. The sensitivity and specificity of NIRF imaging to detect neoplasia in FFT compared to the gold standard of histology was assessed using the Fisher's exact test and was reported with 95% confidence intervals. *P* < *0.05* was considered statistically significant. Statistical analysis was performed using the software GraphPad Prism 9.1.2. (La Jolla, CA, USA; RRID:SCR_002798).

## Results

Seven dogs were presented for the resection of a recurrent STS, 11 with a primary STS and two with a PNST of the sciatic nerve (SI4 Table 1 and 2). The mean preoperative tumor volume calculated based on CT and MRI images was 69 ± 200.5 cm^3^ (SI1 Table 1 and Fig. 1). Angiostamp™ or placebo injection was performed 36 h before surgery in two dogs in the control and one dog in the NIRF group, 6:30 h preoperatively in one patient in the NIRF group and 12 h before surgery in the remaining 16 dogs. The injection of Angiostamp™ did not induce any side effects. In 7/10 dogs in the NIRF group immediate fluorescent uptake in the tumor was visible, one dog showed transcutaneousely visible signal 30 min after injection. The two dogs with a PNST did not display any transdermal signal (SI4 Table 4, Fig. 12).

During the surgical procedure, a NIRF-signal was visible in all tumors of the NIRF group, in 8/10 this was visible transdermally, in 2/10 signal was evident after the lumbar plexus was exposed (SI4 Fig. 1). Resections were adapted based on NIRF-signal in 5/10 cases leading to the resection of neoplastic tissue in four cases that would have been incomplete otherwise (true positives). In one case the NIRF-signal was visible in parts of the scar of the previous resection and margins were adapted. In a second patient residual signal was detected in the tumor bed after resection. In the third patient two fluorescent lymph nodes became visible after resection of the primary tumor was completed. Both nodes were considered metastatic in the histopathologic examination, with one having a sarcoma and one having a carcinoma metastasis. Finally, resection margins were extended based on a fluorescent signal that exceeded the palpable and visible tumor in one dog, and histologic examination confirmed the presence of tumor in this tissue. In the fifth dog the fluorescent signal exceeded the planned margins of tumor resection towards the anus. The resected tissue in this region was highly fluorescent but turned out to be hyperplastic, non-neoplastic circumanal gland tissue (false-positive).

In 1/10 dogs (10%) of the NIRF and 2/10 (20%) dogs in the control group resections were incomplete. All three R1 resections occurred in patients with recurrent STS. Reduction of the 3 cm lateral resection margins to 1 cm around the visible and palpable tumor (control group) or the fluorescent tumor (NIRF group) would have resulted in incomplete resections in five tumors (control group: n = 2/9 (22.2%); NIRF group: n = 3/9 (33.3%)), therefore a minimal margin of 1 cm cannot be recommended, even under NIRF guidance. The most common STS subtype using the veterinary classification were perivascular wall tumors in both groups. In the NIRF group the most common diagnosis based on human sarcoma classification was dermatofibrosarcoma protuberans (SI4 Table 8).

No intraoperative or dye-related complications occurred. Overall postoperative complications related to the surgical procedure or to anesthesia occurred in 11/20 dogs (55%) (SI4 Table 3). Mean follow-up time was 524 ± 330 days (range 1–875) for the NIRF and 607 ± 277 days (range 16–1032) for the control group. Seven out of 20 dogs died (30%), with one sarcoma-related death in the NIRF group. This dog had a veterinary diagnosis of perivascular wall tumor that was re-diagnosed as a rhabdomyosarcoma by the physician-pathologist and a R1 resection, developed a local recurrence 55 days after surgery and died due to presumed abdominal metastatic disease. Another local recurrence was diagnosed in a control group patient with a R1 resection 291 days after surgery (perivascular wall tumor) which was still alive at the last follow-up.

### NIRF image analysis

#### TBR and SBR ratios

Tumor biopsies had a significantly higher SBR (7.4 ± 5.8) compared to the 3 cm margin or tumor bed (*p* = 0.0026; *p* < 0.0001) (Fig. 3a). The TBR at the time of surgery was > 1.5 for all eight patients with superficial STS, four of them reached a TBR ≥ 3 (mean TBR 3.7 ± 2.7). Black table imaging of the native tumor specimen with the skin turned upwards revealed a TBR ≥ 3 in 8/8 patients (mean 6.4 ± 4.9) (Fig. 3b). Notably, while imaging the same specimen, changes in the background or tissue quantity impact MFI, which makes comparison of this parameter across tumors and studies challenging. The tumor bed-to-background ratio was < 1.5 in 5/6 dogs and 1.5 in 1/6 dogs (mean SBR 1.1 ± 0.4). The SBR of the areas with the highest fluorescence in the tumor bed was < 1.5 in 2/6 dogs, 1.5 to 3 in 3/6 dogs and ≥ 3 in 1/6 dogs (mean SBR 2.0 ± 0.8).

**Fig. 4:**
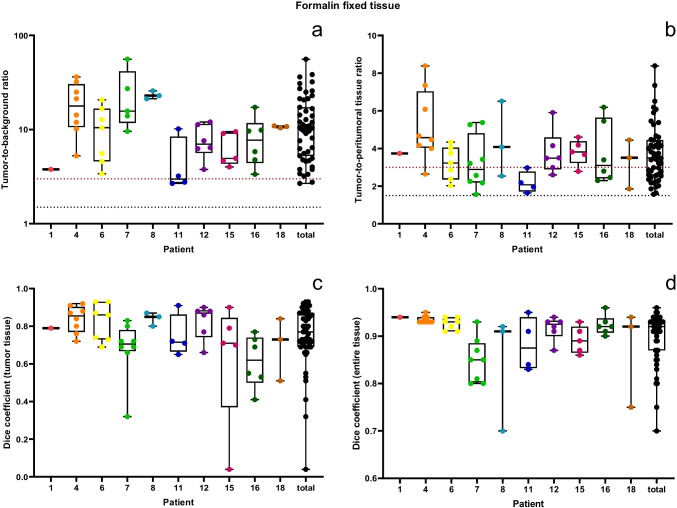
Box plots illustrating the NIRF-signal intensity shown as TBR (**a**), and tumor-to-peritumoral tissue ratio (**b**) and the correspondence of fluorescent area and tumor area (**c**) as well as the correlation between the histological section and the FFT section (**d**) using the Dice coefficient. A Dice coefficient of 1 indicates a perfect match of the areas

The number of FFT sections used to compare the TBR and tumor area versus the NIRF-signal area ranged from one to eight per dog (median 5.5). Fifty-one sections were used to assess NIRF-signal intensity to correlate fluorescent and tumor area (SI4 Table 5). After shape matching, the Dice coefficient for the sample image areas of the histologic section and the image of the FFT section was 0.90 ± 0.05 (Fig. 4d), indicating a high correlation, and therefore successful shape matching. The mean TBR of tumor was 13.5 ± 10.9, while the ratio between tumor and peritumoral non affected tissue (tumor-to-peritumoral tissue ratio) was 3.7 ± 1.5 (Fig. 4a and b). All 10 cases reached a TBR ≥ 3, and 9/10 cases a tumor-to-peritumoral tissue ratio ≥ 3. When comparing the area of NIRF-signal to the histologically confirmed tumor area, the Dice coefficient was 0.75 ± 0.17, indicating a strong correlation (Fig. 4c).

#### Subjective correlation of NIRF-signal and true tumor extent

The NIRF-signal correctly detected presence/absence of tumor in 89.2% (132/148) of the FFT samples (76/132 (57.6%) true positive, 56/132 (42.4%) true negatives). A false positive NIRF-signal was detected in 16/149 sections (10.8%), none of the sections had a false negative NIRF-signal, translating to a sensitivity of 100% (CI 94.8 to 100%) and specificity of 78% (CI 79.2 to 95.2%).

False positive signal was detected in lymph nodes (n = 6), bone (n = 2), testicle (n = 1), circumanal gland (n = 1), and non-affected peritumoral tissue n = 6, that exhibited signs of inflammation (cystic lesion, granulation tissue, reactive muscle fibers, subcutaneous peritumoral inflammatory infiltration) (SI4 Fig. 15).

### Target expression analysis

#### Qualitative and semiquantitative analysis of α_v_β_3_ integrin

Nineteen tumor biopsies were available for analysis. All were α_v_β_3_ integrin positive (> 10% positively stained neoplastic cells) with 17 tumors having over 50% positively stained tumor cells. The detailed α_v_β_3_ integrin staining pattern is described in S1 Fig. 1 and 2. α_v_β_3_ integrin was overexpressed in neoplastic tissue compared to the non-neoplastic tissue in all 19 tumors. The expression score was high in 15/19 (74%), intermediate in 4/19 (21%) and low in 1/19 (5%) tumors.

#### Quantitative analysis of α_v_β_3_ integrin expression

**Fig. 5:**
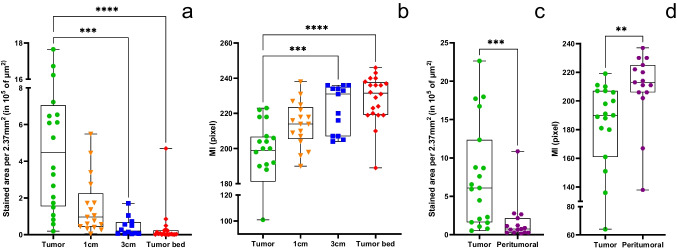
Quantitative analysis of α_v_β_3_ integrin staining in biopsies of the tumor, the tumor bed, the 1 cm and 3 cm margin (**a, b**) as well as in the tumor and peritumoral tissue of tumor biopsies (**c, d**) represented as the α_v_β_3_ integrin-stained area (µm^2^) per 10 high power fields (2.37 mm.^2^) (**a**) and the mean staining intensity (MI) (**b**)

Tumor biopsies showed significantly larger areas and a higher intensity of anti α_v_β_3_ integrin staining, compared to biopsies of the 3 cm margin (area: *p* = 0.0004; MI: *p* < 0.0008) or the tumor bed (area and MI: *p* < 0.0001) (Fig. 5a and b). The stained area and the intensity of staining were higher in the histologically confirmed tumor areas compared to the peritumoral tissue (area: *p* = 0.0004; MI: *p* = 0.0012) (Fig. 5c and d). One third (32%) of the tumors (range: 2–96%) and 7% of the peritumoral tissue (range: 1–46%) were α_v_β_3_ integrin positive. A target selection criteria score of 21/22 was reached for α_v_β_3_ integrin (SI2 Table 1).

There was a strong correlation between the SBR of the tissue biopsies and the area of α_v_β_3_ integrin expression (r = 0.6516; p = 0.0001) and as well as the MI (r = -0.6017; p < 0.0001).

## Discussion

We performed fluorescence-guided surgery using an anti-α_v_β_3_ integrin fluorescent probe for real-time visualization of STS in dogs during tumor resection. Our results demonstrate the safe and successful use of Angiostamp™, resulting in reliable in vivo and ex vivo STS visualization. NIRF based adjustment of the planned resection margins resulted in additional resection of residual tumor in 4/10 cases. We further demonstrated that fluorescence was localized in areas containing tumor as assessed by HE staining and correlated with the expression of α_v_β_3_ integrin, thus we confirmed successful targeting. The overexpression of α_v_β_3_ integrin in canine STS compared to the peritumoral tissue highlights the potential value of this target for resection planning in STS.

Few human and veterinary studies have evaluated the effect of fluorescence-guided surgery on resection success in STS surgery [8, 9, 17, 18, 27–30]. Most of these studies used the untargeted dye ICG [8, 9, 27, 28, 30] with only a few investigating targeted NIRF dyes [17, 18, 29]. While ICG is cheap and FDA approved, untargeted imaging comes with some limitations, including false positive and false negative results. Brooks et al. compared ICG based fluorescence-guided surgery to conventional STS resections in 39 respectively 76 high grade STS [8]. The unexpected R1 rate could be reduced from 25% to 5.1% using ICG [8]. So far, the use of targeted dyes for sarcoma imaging has only been reported sporadically. Steinkamp et al. reported using the bevacizumab-800CW in 15 human STS patients [29]. All tumors could be visualized intraoperatively, and seven tumor-positive margins could be observed during black-table imaging of the tumor specimen. A control group was not included. Two veterinary studies described the use of an anti-α_v_β_3_ integrin dye in 12 feline fibrosarcoma patients [17] and one canine STS [18]. Targeted dyes reliably accumulated in all tumors after intravenous infusion [17, 18, 29], therefore minimizing the number of false negatives. We were able to support this finding in the present study. We also showed that using targeted fluorescence-guided surgery resulted in a histologically confirmed improvement of resection outcome. In the NIRF group four resections were correctly adapted based on fluorescence, resulting in an R0 resection that would otherwise have been R1. E*x vivo* analysis of accuracy resulted in a sensitivity and specificity of 100% and 78%, which compares favorably to what has been described for ICG in humans [28], but not in dogs [30].

Intraoperative NIRF imaging is complex, and various factors including dye performance, imager performance, imaging distance, and angulation as well as impact of environmental light influence the imaging results. A higher contrast between tumor and non-affected tissue is considered fundamental. Ideal cut off signal ratios have been defined to range between 1.5 and 3 [13, 26, 28, 29]. The signal ratios for Angiostamp™ exceeded the upper limit with a mean TBR of 5.0 for transcutaneous imaging, 6.4 for imaging of the excised native specimen, 7.4 for native tissue biopsies and 13.5 for FFT sections. Even the direct comparison of tumor and peritumoral tissue reached a ratio of 3.7. However, the variety of determined TBR at different stages of processing for the same tumor demonstrated that multiple factors impact TBR, which makes comparison of TBR for different dyes across studies challenging. Tumor-to-healthy tissue ratios ranging between 16 (ICG) [30], 14 (Angiostamp™) [17], to 6.5 (ICG) [28], 4.2 (DA364) [18] to 2.0 (bevacizumab-800CW) [29] have been reported for STS. As stated, our results demonstrate that a direct comparison of different dyes is impossible without strict standardization of imaging protocols.

Another factor that influences the results of NIRF imaging is the time point of surgery after dye injection. This issue had been addressed in cats with fibrosarcoma by Wenk et al., determining 36 h being the time interval between injection and surgery to receive the best signal-to-background ratio in cats [17]. In carcinoma bearing mice 16 h had recently been described as ideal [20]. Because no comparable data is available for dogs so far, we operated our first patient 36 h post injection. In this dog we observed already 12 h after injection an adequate tumor contrast that subjectively decreased until 36 h. Thus, the secund patient was operated 6 h post injection. However, a considerable background signal was still visible in this dog. All further patients underwent surgery 12 h post injection (mean 12 h 19 min, range 12 h 05 min to 12 h 45 min) with a good tumor-to-normal tissue contrast (SI4 Fig. 14).

Postoperatively calculated signal ratios quantify a scenario that the surgeon still must subjectively judge during surgery. The discrimination of true signal and artefacts remains challenging, and detection and avoidance of artefacts is essential for correct interpretation. False positive fluorescent signal was observed within the skin, lymph nodes, some bones (ribs, scapula), testicle and circumanal gland in this study (SI4 Fig. 15). The mechanism of dye accumulation in these tissues is unclear, as α_v_β_3_ integrin expression was low to absent in these locations. As all resected lymph nodes in the NIRF group did fluoresce, irrespective of the histological diagnosis, dye uptake as part of normal lymphatic drainage to the sentinel is most likely. However, this remains to be further elucidated. Fluorescence-guided surgery might also be useful to guide metastasectomy, as shown for a single case with lung metastasis in this cohort (SI4 Fig. 14).

However, due to the limited penetration depth of NIRF light preoperative imaging is essential for the detection of metastases and deep tumor lesions. In humans, beside conventional CT and MRI imaging, positron emission tomography (PET) imaging is recommended for patients with certain histotypes or large and high-grade STS. Beside standard FDG PET/CT imaging, serval RGD based radionuclide-labeled tracers had been investigated in different cancer types for tumor and metastases detection, treatment selection for antiangiogenic chemotherapy or monitoring of treatment response [31, 32]. It had been shown that tracer uptake correlates with the expression of α_v_β_3_ integrin [33]. Thus, preoperative RGD based PET molecular imaging of STS patients may be of relevance for patient stratification selecting patients that my benefit from anti-α_v_β_3_ integrin targeted NIRF imaging.

We were able to show that α_v_β_3_ integrin expression correlated with the NIRF-signal intensity, providing evidence for successful targeting in canine STS. While target expression was high in tumor tissue, we observed low, but detectable α_v_β_3_ integrin expression in regions with peritumoral inflammation accompanied by a weak positive NIRF-signal, as previously described [17]. As a precise histological differentiation of sarcoma cells and activated fibroblasts is impossible and resection of peritumoral inflammation is desired, accumulation of Angiostamp™ in these regions is not considered to impair dye performance. α_v_β_3_ integrin reached a target selection criteria score of 21/22, which is superior to the score determined for other potential targets, such as fibroblast activation protein alpha [23]. However, expression pattern and intensity were variable, and due to the limited number of cases and STS subtypes included, we cannot say if this holds true for all STS subtypes.

As this was a pilot study, the number of enrolled patients was small, representing a limitation. Our R1 and local recurrence rate was low compared to published results [3, 4, 12] and compared to results of fluorescence-guided sarcoma resections in humans with a local recurrence of 22% (4/18) [28]. Based on our results, we would need a sample size of 40 cases per group to determine a clinical benefit in terms of a rightfully adapted resection rate. Calculation of the sample size needed to evaluate the impact on survival is not possible, due to the variety of STS subtypes and the fact that very few patients died due to their tumor.

## Conclusion

The results of this pilot study show that the administration of the anti-α_v_β_3_ integrin NIRF dye Angiostamp™ in canine STS patients is safe and results in reliable dye accumulation within STS that allows real-time intraoperative tumor visualization. The high sensitivity of NIRF imaging using Angiostamp™ together with the high NIRF-signal ratios, highlight the potential of this technique and of α_v_β_3_ integrin as target to adequately differentiate STS from healthy tissue. NIRF was especially helpful to pinpoint residual disease in the tumor bed and thereby contributed to an improved resection accuracy.

## Supplementary Information

Below is the link to the electronic supplementary material.

Supplementary file1 (PDF 747 KB)

Supplementary file2(PDF 1318 KB)

Supplementary file3 (PDF 1198 KB)

Supplementary file4 (PDF 4.30 MB)

Supplementary file5 (PDF 4410 KB)

Supplementary file6 (XLSX 120 KB)

Supplementary file7 color fused, IR and visible videos of the tumor specimen of Dog6 (MP4 71791 KB)

Supplementary file8 color fused, IR and visible videos of the tumor specimen of Dog6 (MP4 2644 KB)

Supplementary file9 color fused, IR and visible videos of the tumor specimen of Dog6 (MP4 78618 KB)

Supplementary file10 color fused, IR and visible video of the surgery of Dog6 (MP4 40654 KB)

Supplementary file11 color fused, IR and visible video of the surgery of Dog6 (MP4 6877 KB)

Supplementary file12 color fused, IR and visible video of the surgery of Dog6 (MP4 45363 KB)

Supplementary file13 color fused, IR and visible video of the surgery of Dog7 (MP4 35920 KB)

Supplementary file14 color fused, IR and visible video of the surgery of Dog7 (MP4 37975 KB)

Supplementary file15 color fused, IR and visible video of the surgery of Dog7 (MP4 3.89 MB)

## Data Availability

The datasets generated and analyzed during the current study are available in the Harvard Dataverse repository, 10.7910/DVN/VL73MV.
