# Supplementary material for: Evaluation of a targeted anti-αvβ3 integrin near-infrared fluorescent dye for fluorescence-guided resection of naturally occurring soft tissue sarcomas in dogs
Source: Eur J Nucl Med Mol Imaging. 2024 Oct 22;52(3):1137–48. doi: 10.1007/s00259-024-06953-x (PMC11754361; doi:10.1007/s00259-024-06953-x)
Supplement: Supplementary file 1 — Supplementary file1 (PDF 747 KB) [file 259_2024_6953_MOESM1_ESM.pdf]

Supplementary Information (SI) 1

Diagnostic imaging

Evaluation of a targeted anti-$\alpha_v\beta_3$ integrin near-infrared fluorescent dye for fluorescence-guided resection of naturally occurring soft tissue sarcomas in dogs.

European Journal of Nuclear Medicine and Molecular Imaging

Patricia Beer[1*], Paula Grest[2], Christiane Krudewig[2], Chris Staudinger[3], Stefanie Ohlerth[3], Carla Rohrer Bley[4], Armin Jarosch[5], Houria Ech-Cherif[6], Enni Markkanen[6], Brian Park[1], Mirja Christine Nolff[1]

[1]Clinic for Small Animal Surgery, University Animal Hospital, Vetsuisse Faculty, University of Zurich, Zurich, Switzerland

[2]Institute of Veterinary Pathology, Vetsuisse Faculty, University of Zurich, Zurich, Switzerland

[3]Clinic for Diagnostic Imaging, University Animal Hospital, Vetsuisse Faculty Zurich, University Zurich, Zurich, Switzerland

[4]Division of Radiation Oncology, University Animal Hospital, Vetsuisse Faculty Zurich, University Zurich, Zurich, Switzerland

[5]Department of Pathology, Charité-Universitätsmedizin Berlin, Corporate Member of Freie Universität Berlin and Humboldt-Universität zu Berlin, Berlin, Germany

[6]Institute of Veterinary Pharmacology and Toxicology, Vetsuisse Faculty, University of Zurich, Zurich, Switzerland

*corresponding author: pbeer@vetclinics.uzh.ch

1. Materials and methods

1.1. Image acquisition

Based on current gold standards in veterinary medicine, each patient underwent a computed tomography (CT) scan of the tumor and the thorax for staging preoperatively. CTs were completed using a 16-row CT scanner (Brilliance CT, Philips AG, Zurich, Switzerland) or a 64 x 2 row CT scanner (IQon Spectral CT, Philips AG, Zurich, Switzerland) with the following scan parameters: slice thickness 1 mm, 120 kV, 180 mAs (thorax) or 200 mAs (abdomen/pelvis), pitch 0.6. After completion of a native scan, an iodinated, low-osmolar contrast agent (Accupaque 350; 350 mg iodine/mL) was injected at a dosage of 2 ml/kg body weight and an injection rate of 2 ml/s (Accutron CT-D Medtron Injector, SMD Medial AG, St. Gallen, Switzerland). Bolus tracking with a threshold of 100 HU was performed. The tracker was placed within the aortic arch in masses localized on the front limbs, thorax or head and neck region, within the abdominal aorta for imaging abdominal/pelvic tumors, or within the external iliac artery in tumors of the hind limbs. The scan was repeated 40-60 s after the first post-contrast CT scan. All images were stored in the clinical picture archiving and communication system (PACS) in Digital Imaging and Communications in Medicine (DICOM) format.

Four dogs were already presented with CT scans performed prior to presentation, these scans were not repeated for ethical (radiation safety, general anesthesia) and financial reasons. One case with a peripheral nerve sheath tumor (PNST) of the sciatic nerve had thoracic radiographs (right and left lateral) and a magnetic resonance imaging (MRI) study of the tumor region instead of the CT (3 Tesla Philips Ingenia, Philips AG, Zurich, Switzerland).

1.2. Image evaluation

A board-certified ECVDI radiologist (S.O.) and a resident in diagnostic imaging (C.S.) evaluated the following criteria on pre- and postcontrast CT scans in all tumors (adapted following Flemming et al. (2019) [1]): pre-contrast delineation (completely surrounded by fatty tissue, partial contact with the skin or muscles, within a facial plane or muscle), tumor shape (spindle-shaped, ovoid, multilobulated), post-contrast tumor delineation (no, poor, fair, good), uniformity of enhancement (homogenous, heterogenous) and involvement of muscles or neuro-vascular structures, presence of tumor extensions, rim enhancement, feeding vessels, flat soft tissue connecting two or more neoplastic nodules, tumor mineralization, adjuvant lesions, peritumoral inflammation or bone lysis (yes, no).

1.3. Tumor volumetry

Tumor size (maximum length, width, height) determined at the time of histologic sample evaluation was compared to postcontrast CT images. Tumor volume was calculated using linear measurements of the maximum tumor dimensions and the rotation ellipsoid formula ($\pi/6 \times$ height $\times$ width $\times$ depth) [2]. In addition, tumor volume was measured with an open-source software application for medical image computing (3D Slicer, version 5.4.0) [3]. After the CT study was reduced to the tumor region, the tumor segment was delineated using the Segment Editor. Whenever possible, contouring was done in one plane

if needed, the other two planes were included. The volume of the segment was then calculated using the "Segment Statistics" module.

1.4. Statistical analysis

Statistical analysis was performed using the software GraphPad Prism 9.1.2. (La Jolla, CA, USA; RRID:SCR_002798). As the data was not normally distributed a Friedman Test was used to assess differences between tumor volume measurements. Spearman correlation was performed to analyze the correlation between the tumor volumes measured with the different techniques. A Kruskal-Wallis test and a Dunn's post-hoc analysis was used to assess differences between the tumor volume of the different tumor grades. A p-value of < 0.05 was set as level for significance.

2. Results

In total, CT examinations of 19 tumors and an MRI scan of one tumor were available for assessment. Thoracic imaging revealed the presence of a histologically confirmed lung metastasis in one dog. Two additional dogs had lung nodules visible at the time of staging: in one, the lung nodules disappeared after anthelmintic treatment, the other dog died due to suspected metastatic disease without a histopathological examination.

Tumor-specific findings after contrast application are listed in table 1 and the supplemental excel file (raw data).

Table 1: Selected post-contrast CT imaging features and tumor volume measurements of canine STS grouped by tumor grade.

**Features**	**Results**				
Tumor grade	**Grade 1**	**Grade 2**	**Grade 3**	**No grade**	**No grade PNST**
Number of patients	n=10	n=4	n=3	n=1	n=2
Tumor extensions	6 (66.7%, 6/9)	3 (100%, 3/3)	2 (100%, 2/2)	1	0
Multilobulated shape	2 (20%, 2/10)	1 (25%, 1/4)	3 (100%, 3/3)	1	0
Rim enhancement	4 (40%, 4/10)	2 (50%, 2/4)	2 (66.6%, 2/3)	1	0
Heterogenous contrast uptake	6 (60%, 6/10)	2 (50%, 2/4)	3 (100%, 3/3)	1	0
Homogenous contrast uptake	4 (40%, 4/10)	2 (50%, 2/4)	0 (0%, 0/3)	1	2 (100%, 2/2)
CT tumor volume ellipsoid in cm^3 (mean and range)	26.3 (n=8) (0.6-153.3) 22.7 (n=10) (0.6-153.3)	63.4 (4.3-111.9)	306.3 (17.1-845.0)	7.3	
CT tumor volume 3D slicer in cm^3 (mean and range)	25.7 (n=8)* (0.6-161.97) 22.1 (n=10)** (0.6-161.9)	47.0 (3.4-73.1)	318.9 (16.8-904.9)	8.5	2.9 (2.7-3.0)
Pathology FF tumor volume in cm^3 (mean and range)***	13.7 (n=8) (0.1-80.1)	40.4 (2.1-81.3)	386.8 (17.3-1123.1)	9.0	

*Mean tumor volume for the 7 tumors with available macroscopic tumor volume measurements.

**Mean tumor volume for all 10 grade 1 STS.

*** The tumor volume of the formalin fixated (FF) specimen was calculated by measuring the length, height, and width of the macroscopically visible tumor during specimen trimming. Visual tumor margin delineation by the pathologist was sufficient at the time of tumor trimming to carry out measurements in 15/17 tumors.

Mean tumor volume for the 16 tumors measured by all three methods was 90.0±276.8 cm^3 for the macroscopic estimation, 86.9±207.1 cm^3 for the CT based ellipsoid measurements and 84.9±222.6 cm^3 for the 3D slicer measurements. Direct comparison did not reveal a significant difference between measurement method ($p > 0.2792$), and all methods showed strong correlation ($r=0.9329$, $r=0.9478$, $r=0.9411$) (Fig. 1a and 2), indicating accurate detection of tumor dimension in postcontrast CT across all tumor types and grades ($p > 0.1089$).

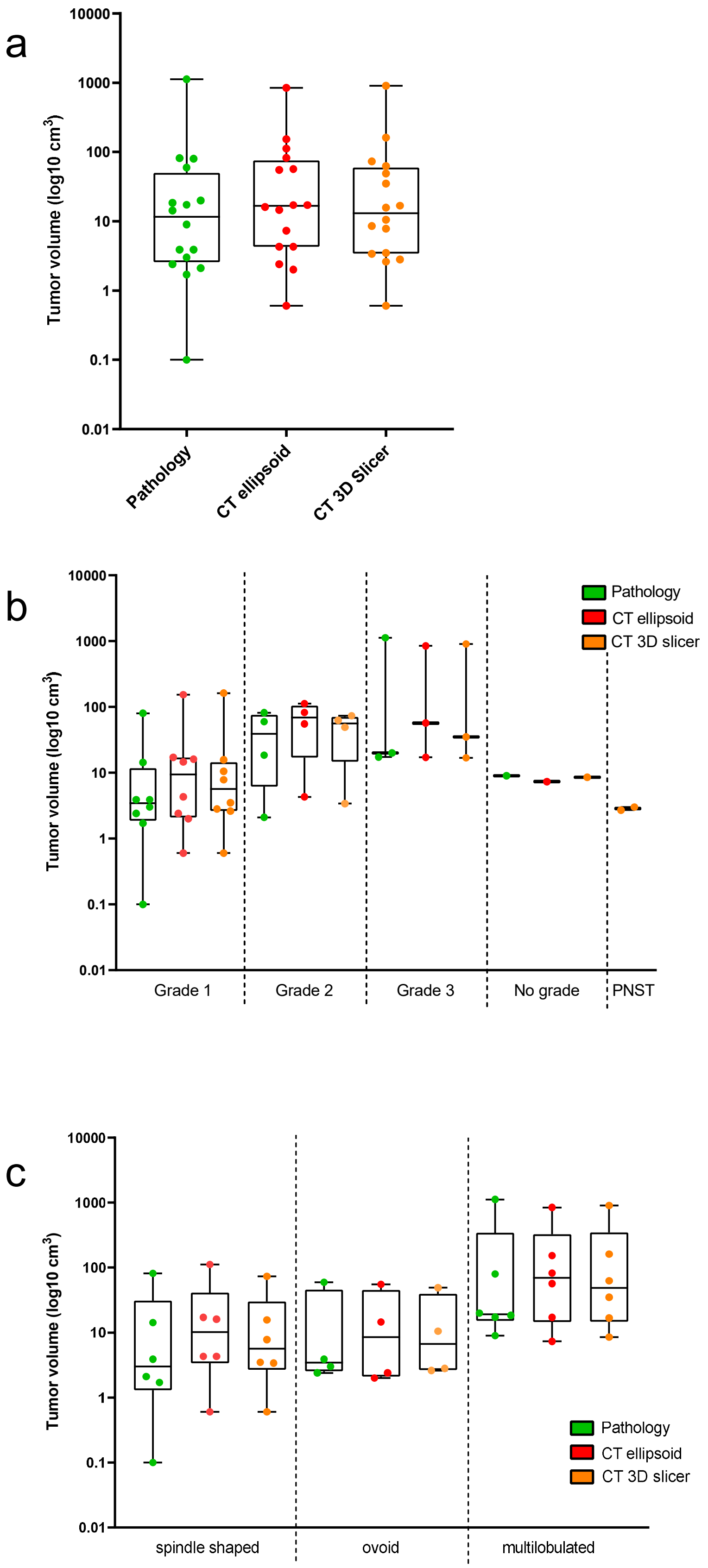

Fig. 1 Box plots representing the measured tumor volumes using the three measurement techniques (a) in 16 STS, (b) after being classified according to the tumor grades with ungraded peripheral nerve sheath tumors (PNST) being listed separately and (c) after classification by their shape.

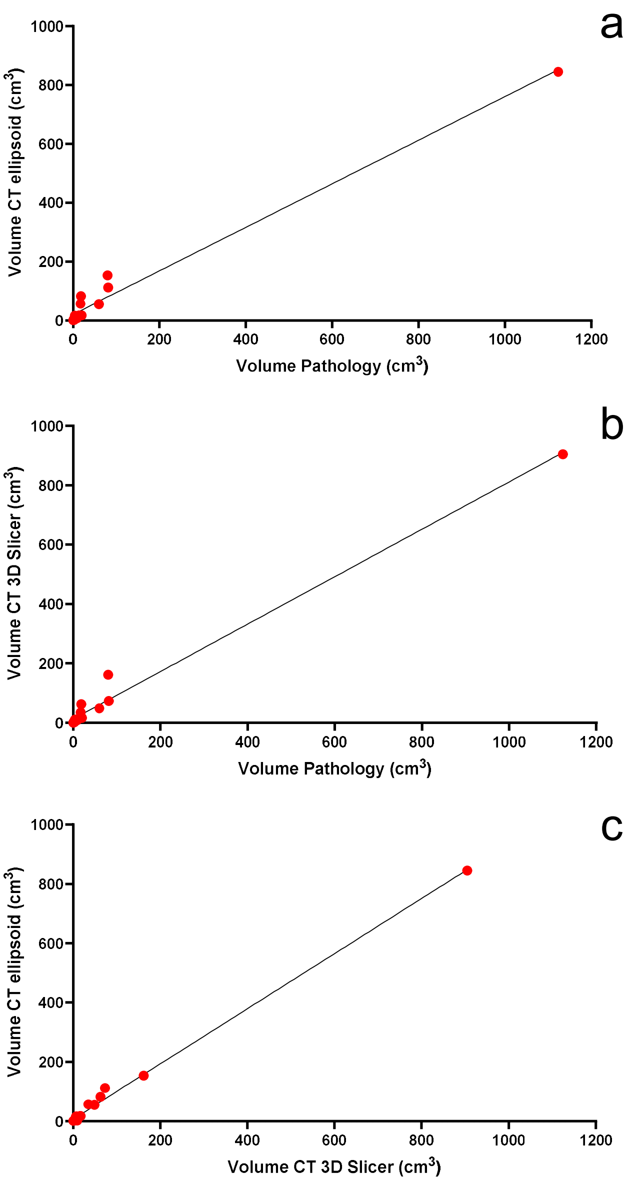

Fig. 2 Scatter plots demonstrating the distribution of macroscopically measured tumor volumes versus CT based tumor volume calculated using the ellipsoid formula (a) or the 3D slicer (b). (c) illustrates the relationship between the CT based tumor volume measurements. There was a strong positive correlation between the tumor volume measured during the pathological examination and the CT based tumor volume calculated using the ellipsoid formula ($r=0.9329$, $p<0.0001$) (a), between the macroscopic tumor volume and the CT based tumor volume calculated using the 3D slicer ($r=0.9478$, $p<0.0001$) (b), and between the tumor volume of the two CT based measurement methods ($r=0.9411$, $p<0.0001$) (c).

.

3. References

1. Fleming K, Nemanic S, Löhr CV, Terry J, Milovancev M. CT angiography and MRI imaging features do not predict the tumor type and grade of feline injection site sarcoma. Vet Radiol Ultrasound. 2019;60:668-79. doi:10.1111/vru.12807.
2. Begg, A, C. The growth rate of tumours. In: Steel, G., Gordon, editors. Basic Clinical Radiobiology. 3 ed: Edward Arnold, London; 2002. p. 8-22.
3. Fedorov A, Beichel R, Kalpathy-Cramer J, Finet J, Fillion-Robin JC, Pujol S, et al. 3D Slicer as an image computing platform for the Quantitative Imaging Network. Magn Reson Imaging. 2012;30:1323-41. doi:10.1016/j.mri.2012.05.001.
