# Supplementary material for: Evaluation of a targeted anti-αvβ3 integrin near-infrared fluorescent dye for fluorescence-guided resection of naturally occurring soft tissue sarcomas in dogs
Source: Eur J Nucl Med Mol Imaging. 2024 Oct 22;52(3):1137–48. doi: 10.1007/s00259-024-06953-x (PMC11754361; doi:10.1007/s00259-024-06953-x)
Supplement: Supplementary file 2 — Supplementary file2(PDF 1318 KB) [file 259_2024_6953_MOESM2_ESM.pdf]

Supplementary Information (SI) 2

Immunohistochemical analysis of $\alpha_v\beta_3$ integrin expression

Evaluation of a targeted anti-$\alpha_v\beta_3$ integrin near-infrared fluorescent dye for fluorescence-guided resection of naturally occurring soft tissue sarcomas in dogs.

European Journal of Nuclear Medicine and Molecular Imaging

Patricia Beer[1*], Paula Grest[2], Christiane Krudewig[2], Chris Staudinger[3], Stefanie Ohlerth[3], Carla Rohrer Bley[4], Armin Jarosch[5], Houria Ech-Cherif[6], Enni Markkanen[6], Brian Park[1], Mirja Christine Nolff[1]

[1]Clinic for Small Animal Surgery, University Animal Hospital, Vetsuisse Faculty, University of Zurich, Zurich, Switzerland

[2]Institute of Veterinary Pathology, Vetsuisse Faculty, University of Zurich, Zurich, Switzerland

[3]Clinic for Diagnostic Imaging, University Animal Hospital, Vetsuisse Faculty Zurich, University Zurich, Zurich, Switzerland

[4]Division of Radiation Oncology, University Animal Hospital, Vetsuisse Faculty Zurich, University Zurich, Zurich, Switzerland

[5]Department of Pathology, Charité-Universitätsmedizin Berlin, Corporate Member of Freie Universität Berlin and Humboldt-Universität zu Berlin, Berlin, Germany

[6]Institute of Veterinary Pharmacology and Toxicology, Vetsuisse Faculty, University of Zurich, Zurich, Switzerland

*corresponding author: pbeer@vetclinics.uzh.ch

1. Materials and Methods

1.1. Cryoembedding of tissue biopsies

Tissue freezing was performed using the PrestoCHILL (Milestone, Italy). A drop of embedding medium (MCC) was put on a dispensing slide covered by a paper disc, where the samples were placed. The paper was then placed into the bottom of the mold. The mold was filled up with embedding medium. The chick and the heat extractor were placed on the mold. The samples were frozen after 80 seconds and covered by the paper disk. A chuck was placed in the cryostat and the block was trimmed until the paper was cut away. A tissue slice was used for routine hematoxylin and eosin (HE) staining, and one was used for immunohistochemistry (IHC).

1.2. Immunohistochemical staining protocol for anti-$\alpha_v\beta_3$ integrin

IHC was performed on 8 µm thick sections of optimal cutting temperature (OCT) tissue blocks using the validated primary anti-integrin $\alpha_v\beta_3$ antibody clone LM609 (MAB1976; RRID:AB_2925190) diluted 1:400. Tissue sections were mounted on positively-charged slides (SuperFrost Plus-OTs, Thermo Fisher Scientific), fixed in acetone for 10 min and rinsed with wash-buffer TBS (Dako 3006). Slides were incubated in 1:400 primary antibody and antibody diluent (Dako S2022) for 1 hour in a wet chamber at room temperature, followed by a washing step in wash-buffer TBS (Dako 3006). Slides were put into the autostainer. Peroxidase blocking (peroxidase blocking buffer, Dako S2023) was performed for 10 min at room temperature, followed by incubation with the secondary antibody (Envision+System HRP Mouse (Dako K4001)) for 30 min at room temperature. Between those steps, slides were rinsed with wash-buffer TBS (Dako 3006). For visualization, the DAB Detection Kit (Dako K3468) was used for an incubation time of 10 min at room temperature followed by rinsing with tap water. All sections were counterstained with hematoxylin (modified to Gill 2) (Merck 1.05175.0500) for 2 seconds, rinsed with tap water, dehydrated and cover slipped.

1.3. Analysis of $\alpha_v\beta_3$ integrin staining

Qualitative and semiquantitative analysis of $\alpha_v\beta_3$ integrin staining was performed by two investigators (P.G., P.B.). Quantitative analysis of IHC stained slides for $\alpha_v\beta_3$ integrin expression was performed using an automated histomorphometry software Visiopharm® (Hoersholm, Denmark) as described in Beer et al. 2023 [1]. A Target Selection Criteria (TASC) score for anti-$\alpha_v\beta_3$ integrin was assessed [23, 24]. Criteria I, V, VI and IIV were evaluated based on published literature. Judgment of criteria II and IV was performed using the results of the qualitative IHC expression analysis. For criterium III, the tumor-to-normal tissue ratio of the $\alpha_v\beta_3$ integrin expression, the ratio of the $\alpha_v\beta_3$ integrin positive area in tumor samples in relation to the positively stained area in tumor bed samples was used.

2. Results

Biopsies taken from the tumor as well as intra- and peritumoral endothelial cells of venous and arterial blood vessels showed consistent and strong positivity for $\alpha_v\beta_3$ integrin. Endothelial $\alpha_v\beta_3$ integrin expression contributed considerably to the positivity of the whole tumor tissue in some cases (Fig. 1). The tunica media of arteries showed inconsistent $\alpha_v\beta_3$ integrin positivity, equally to other cell types and structures such as fibrocytes, fibroblasts, inflammatory cells, perifollicular cells, peripheral nerve or collagen fibres (Fig. 2). Lymphatic tissue and cells of the circumanal gland did not show a $\alpha_v\beta_3$ integrin positivity, although lymph nodes and circumanal gland tissue were highly fluorescent.

A marked inter-tumoral heterogeneity of the integrin positive areas and the intensity of staining ranging from strong positivity to complete absence of staining was observed in the tumor tissue samples. The expression score for $\alpha_v\beta_3$ integrin is displayed in S1 4 Table 2.

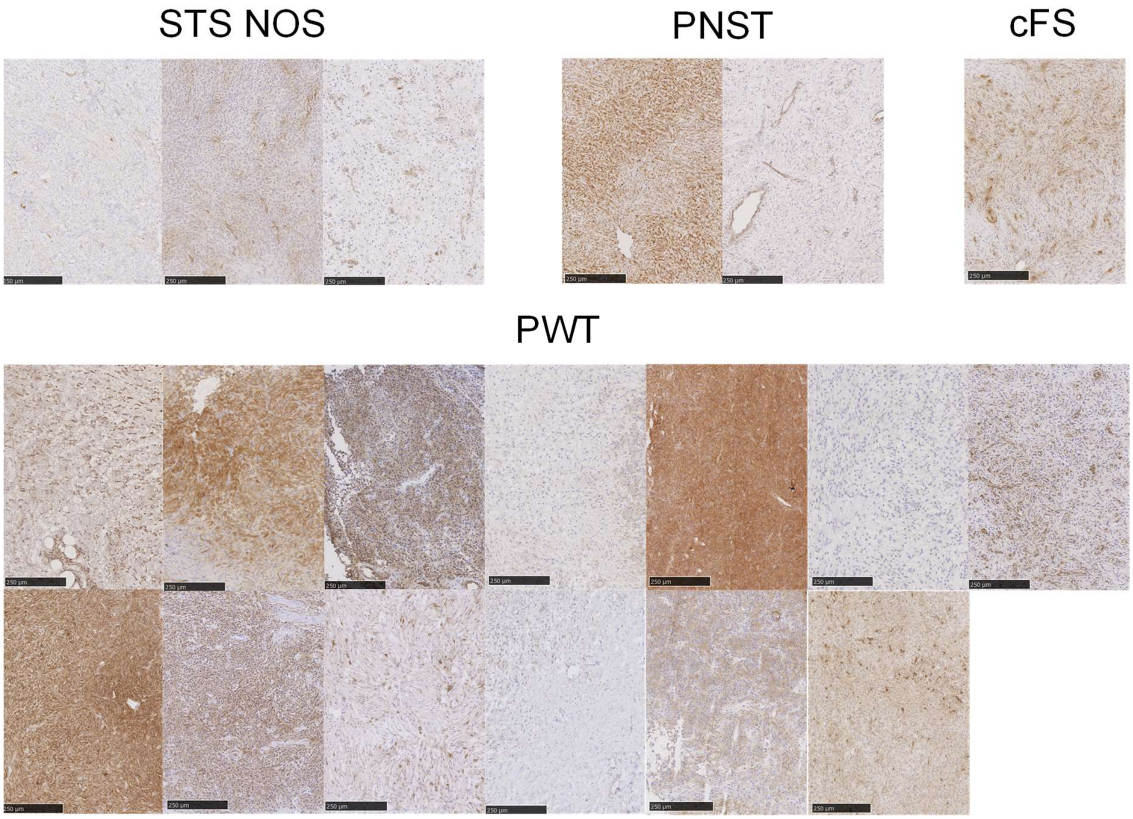

Fig. 1 Anti-$\alpha_v\beta_3$ integrin-stained tumor sections of three STS not otherwise specified (STS NOS), two peripheral nerve sheath tumors (PNST), one canine fibrosarcoma (cFS) and 13 perivascular wall tumors (PWT) of 19 dogs. The $\alpha_v\beta_3$ integrin expression in the tumors ranged from low to high. In several tumors, a strong positivity of intratumoral blood vessels was observed (scale bar 250 µm).

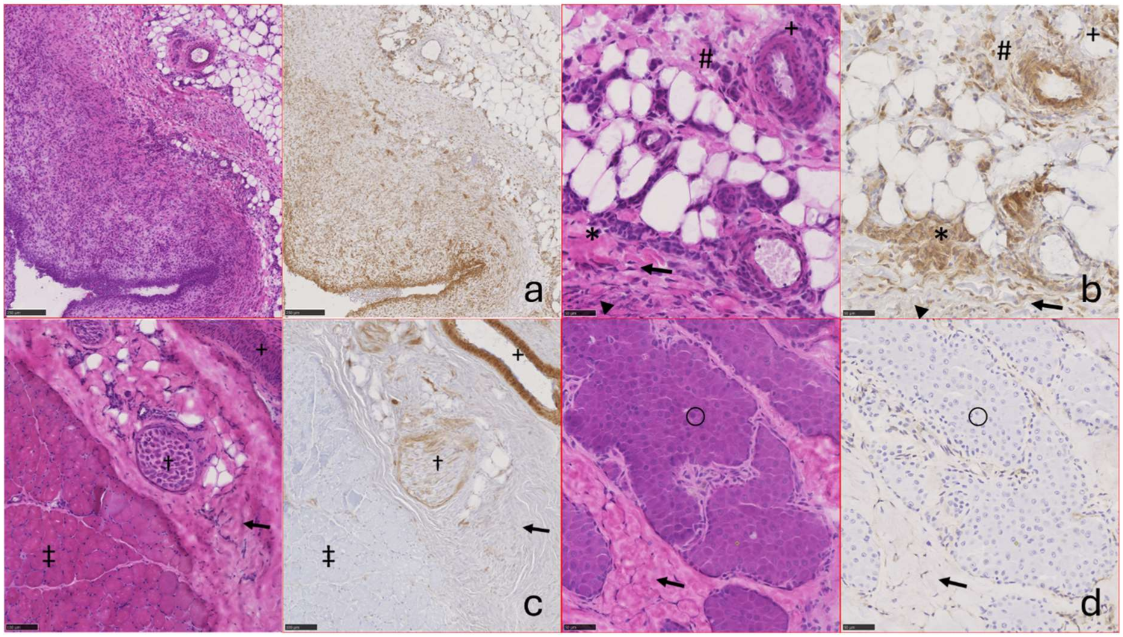

Fig. 2 Hematoxylin and eosin stained and corresponding anti-$\alpha_v\beta_3$ integrin-stained frozen tissue sections of non-neoplastic tissue (magnification (a) 20x, (b and d) 40x). (a and b) Region of granulation tissue in a recurrent STS of the thoracic wall (case 6) with intermediate to strong positivity for $\alpha_v\beta_3$ integrin. The asterisk (*) marks a region of $\alpha_v\beta_3$ integrin positive immune cells that are also infiltrating the adipose tissue. The collagen fibres (arrow) are unstained while the fibrocytes are positive (arrowhead). The hashtag (#) marks a newly formed blood vessel with $\alpha_v\beta_3$ integrin positive endothelial cells. Likewise endothelial and smooth muscle cells of the tunica media of the artery (+) show $\alpha_v\beta_3$ integrin expression. (c) The (+) marks the $\alpha_v\beta_3$ integrin positive tunica media and externa of a vein, (†) a positive peripheral nerve and (‡) negative skeletal muscle fibres. (d) shows $\alpha_v\beta_3$ integrin negative healthy circumanal gland tissue (circle) surrounded by connective tissue (arrow) with some positive fibroblasts.

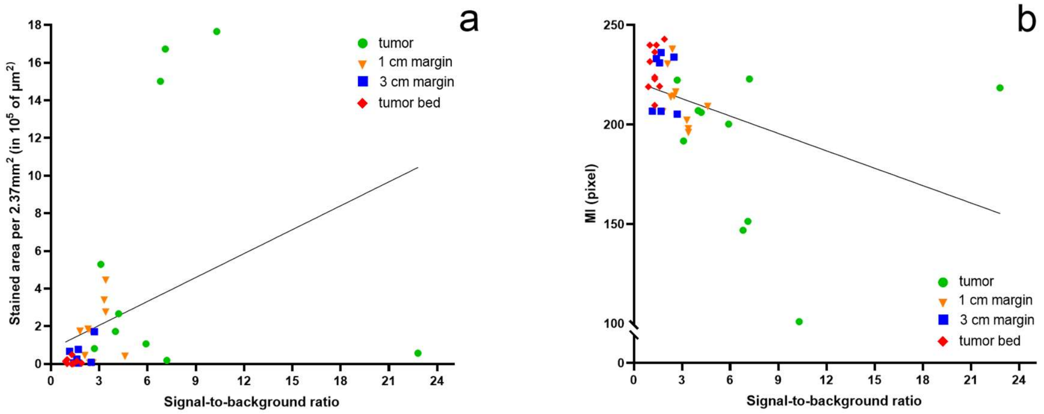

Fig. 3 Scatter plots demonstrating the distribution of signal-to-background ratios (SBR) with the $\alpha_v\beta_3$ integrin-stained area (µm^2) per 10 high power fields (2.37 mm^2) (a) and the mean staining intensity (MI) (b).

Table 1: Target selection criteria scoring (TASC) [1, 3, 4] of $\alpha_v\beta_3$ integrin based on the results of the immunohistochemical expression analyses and knowledge derived from literature.

**Criteria**		**Description of the scoring system**	**Max. score**	**$\alpha_v\beta_3$ integrin - scoring**
I	Extracellular protein localization	Receptor bound to cell surface = 5<br>In close proximity of the tumor cell = 3	5	5 [5, 6]
II	Diffuse upregulation through tumor tissue	Staining ≥50 % of tumor cells in the majority (>50 %) of patients = 4<br>Staining <50 % of tumor cells and/or in <50 % of patients = 0	4	4 (17/19 (90%))
III	Tumor to normal tissue ratio	T/N > 10 = 3<br>T/N ≤ 10 = 0	3	2 (15/19 (79%))
IV	Percentage of upregulation in patients	≥ 90 % = 6<br>70–89 % = 5<br>50–69 % = 3<br>10–49 % = 0	6	6 (19/19 (100%))
V	Previous imaging success *in vivo*	Yes = 2 (including NIR imaging, PET/CT, MRI and other imaging modalities)	2	2 [7-9]
VI	Enzymatic activity	Yes =1	1	0
VII	Target-mediated internalization	Yes =1	1	1 [6]
**Total**			**22**	**21**

3. References

1. Beer P, Pauli C, Haberecker M, Grest P, Beebe E, Fuchs D, et al. Cross-species evaluation of fibroblast activation protein alpha as potential imaging target for soft tissue sarcoma: a comparative immunohistochemical study in humans, dogs, and cats. Front Oncol. 2023;13:1210004. doi:10.3389/fonc.2023.1210004.
2. Meuten D. Appendix: Diagnostic Schemes and Algorithms. In: DJ M, editor. Tumors in Domestic Animals; 2016. p. 942-78.
3. van Oosten M, Crane LM, Bart J, van Leeuwen FW, van Dam GM. Selecting Potential Targetable Biomarkers for Imaging Purposes in Colorectal Cancer Using TArget Selection Criteria (TASC): A Novel Target Identification Tool. Transl Oncol. 2011;4:71-82. doi:10.1593/tlo.10220.
4. de Gooyer JM, Versleijen-Jonkers YMH, Hillebrandt-Roeffen MHS, Frielink C, Desar IME, de Wilt JHW, et al. Immunohistochemical selection of biomarkers for tumor-targeted image-guided surgery of myxofibrosarcoma. Sci Rep. 2020;10:2915. doi:10.1038/s41598-020-59735-4.
5. Beauvais DM, Ell BJ, McWhorter AR, Rapraeger AC. Syndecan-1 regulates alphavbeta3 and alphavbeta5 integrin activation during angiogenesis and is blocked by synstatin, a novel peptide inhibitor. J Exp Med. 2009;206:691-705. doi:10.1084/jem.20081278.
6. Danhier F, Le Breton A, Préat V. RGD-based strategies to target alpha(v) beta(3) integrin in cancer therapy and diagnosis. Mol Pharm. 2012;9:2961-73. doi:10.1021/mp3002733.
7. Wenk CH, Ponce F, Guillermet S, Tenaud C, Boturyn D, Dumy P, et al. Near-infrared optical guided surgery of highly infiltrativ αvβ3e fibrosarcomas in cats using an anti-αvß3 integrin molecular probe. Cancer Lett. 2013;334:188-95. doi:10.1016/j.canlet.2012.10.041.
8. Mery E, Golzio M, Guillermet S, Lanore D, Le Naour A, Thibault B, et al. Fluorescence-guided surgery for cancer patients: a proof of concept study on human xenografts in mice and spontaneous tumors in pets. Oncotarget. 2017;8:109559-74. doi:10.18632/oncotarget.22728.
9. Favril S, Brioschi C, Vanderperren K, Abma E, Stock E, Devriendt N, et al. Preliminary safety and imaging efficacy of the near-infrared fluorescent contrast agent DA364 during fluorescence-guided surgery in dogs with spontaneous superficial tumors. Oncotarget. 2020;11:2310-26. doi:10.18632/oncotarget.27633.
