# Supplementary material for: Evaluation of a targeted anti-αvβ3 integrin near-infrared fluorescent dye for fluorescence-guided resection of naturally occurring soft tissue sarcomas in dogs
Source: Eur J Nucl Med Mol Imaging. 2024 Oct 22;52(3):1137–48. doi: 10.1007/s00259-024-06953-x (PMC11754361; doi:10.1007/s00259-024-06953-x)
Supplement: Supplementary file 3 — Supplementary file3 (PDF 1198 KB) [file 259_2024_6953_MOESM3_ESM.pdf]

Supplementary Information (SI) 3

Antibody validation

Evaluation of a targeted anti-$\alpha_v\beta_3$ integrin near-infrared fluorescent dye for fluorescence-guided resection of naturally occurring soft tissue sarcomas in dogs.

European Journal of Nuclear Medicine and Molecular Imaging

Patricia Beer[1*], Paula Grest[2], Christiane Krudewig[2], Chris Staudinger[3], Stefanie Ohlerth[3], Carla Rohrer Bley[4], Armin Jarosch[5], Houria Ech-Cherif[6], Enni Markkanen[6], Brian Park[1], Mirja Christine Nolff[1]

[1]Clinic for Small Animal Surgery, University Animal Hospital, Vetsuisse Faculty, University of Zurich, Zurich, Switzerland

[2]Institute of Veterinary Pathology, Vetsuisse Faculty, University of Zurich, Zurich, Switzerland

[3]Clinic for Diagnostic Imaging, University Animal Hospital, Vetsuisse Faculty Zurich, University Zurich, Zurich, Switzerland

[4]Division of Radiation Oncology, University Animal Hospital, Vetsuisse Faculty Zurich, University Zurich, Zurich, Switzerland

[5]Department of Pathology, Charité-Universitätsmedizin Berlin, Corporate Member of Freie Universität Berlin and Humboldt-Universität zu Berlin, Berlin, Germany

[6]Institute of Veterinary Pharmacology and Toxicology, Vetsuisse Faculty, University of Zurich, Zurich, Switzerland

*corresponding author: pbeer@vetclinics.uzh.ch

1. Materials and Methods

1.1. Cells and cell culture

Canine soft tissue sarcoma-derived primary cell lines of Dog1, Dog5 and Dog8 were established as described in [1] and grown under standard culture conditions (37°C, 5% CO_2) in 10 cm cell culture dishes in Gibco™ DMEM low glucose pyruvate medium (Fisher scientific, 21885-025) supplemented with 20% fetal bovine serum (FBS; Corning, 35-010-CV), 1% antibiotic/antimycotic solution (Sigma-Aldrich, A5955) and 1% ITS (Corning, 25-800-CR). Epithelial COSCC cells (derived from a canine oral squamous cell carcinoma) were grown in medium Gibco™ DMEM high glucose pyruvate media (Fisher scientific, 41966-029) supplemented with 15% FBS, 1% antibiotic/antimycotic solution and 1% MEM NEA (Gibco, 11140), as previously described [2]. For passaging, cells were detached from the plates using 2 ml of 0,25% Trypsin (Trypsin Gibco-15090-046) supplemented with 0.02% EDTA (EDTA Biochemica, Panreac Application A1103, 1000), incubated for 5 min at 37 °C and neutralized using the respective complete standard medium. Detached cells in suspension were further processed and used for RT-qPCR analysis or seeded on cover slides for subsequent immunofluorescence (IF) imaging.

1.2. Reverse Transcription quantitative PCR (RT-qPCR)

Total RNA was isolated from cell pellets using the RNeasy® Mini Kit (Qiagen, 74106) according to the manufacturer's protocol. The isolated RNA was quantified, and quality checked using NanoDrop. For each sample, 1 µg of RNA was reverse transcribed using the iScript cDNA Synthesis Kit (Bio-Rad, 1708891) according to the manufacturer's protocol on a LabCycler (SensoQuest). The cDNA was diluted 1:5 with nuclease-free water before use in RT-qPCR. Quantitative reverse transcription PCR (qRT-PCR) was performed using the KAPA SYBR® FAST One-Step qRT-PCR Kit in a total volume of 10 µl in duplicates on the CFX384 Touch™ Real-Time PCR detection system (Bio-Rad). Primers for canine integrin alpha 5 and integrin beta 3 were designed using Primer3 based on the mRNA reference sequences (XM_038674953.1 and XM_038446753.1) from NCBI (https://www.ncbi.nlm.nih.gov/). All following primers were ordered from Microsynth (Balgach, Switzerland): cITGAV-fw: 5'-GACCTCCAACCCCATGAGAA-3', cITGAV-rev: 5'-AGGTAATGTCCGTGTGAGCA-3', cITGB3-fw: 5'-TGGCAGTCTTGTGTCAGCTA-3', cITGB3-rev: 5'-ACTGGATTGAGGTGATGGCA-3', cGAPDH-fw: 5'- CATCACTGCCACCCAGAAG-3', cGAPDH-rev: 5'-CAGTGAGCTTCCCGTTCAG-3', c-bActin-fw: 5'-TTGCCGACAGGATGCAGAA-3', c-bActin-rev: 5'-GCTCAGGAGGAGCAATGATCTT-3'. Quantification of gene expression was performed using the comparative CT method, values were normalized against GAPDH and c-bActin, and COSCC cells as the control, and results were expressed as fold change in mRNA levels over COSCC cells. Each experiment was independently repeated two times, and data are expressed as individual data points and mean ± SD.

1.3. Immunofluorescence for $\alpha_v\beta_3$ integrin

Cells were grown in 6-well plates on sterile 14-mm glass coverslips and fixed with 4% paraformaldehyde (pH 8.0) in PBS for 15 min at room temperature. After washing 3 x 5 min in PBS, permeabilization was performed using 0.2% Triton X-100 in PBS for 5 min at room temperature, followed by blocking for 30 min in 1% bovine serum albumin (BSA)–PBS. Samples were incubated with primary antibody anti-integrin $\alpha_v\beta_3$ antibody, clone LM609 (MAB1976; RRID:AB_2925190) diluted to a concentration of 5ug/ml in 1% BSA–PBS and for 1h at room temperature, washed 3 x with PBS, and subsequently incubated for 30 min with the secondary antibody (1:400 of Alexa FluorTM 488 green (A11029) diluted in 1% BSA–PBS supplemented with 600nM DAPI solution) at room temperature in the dark. After washing 3 x 5 min with PBS, cover slips were mounted on glass slides using ProLong Gold Antifade Mountant (Invitrogen) and dried overnight. Fluorescence microscopy was performed using the Leica DMI6000 Bat using 20x magnification.

2. Results

Higher expression levels of integrin α_v and integrin β_3 mRNA in sarcoma cells from Dog1, Dog5 and Dog8 in comparison to COSCC cells were validated using RT-qPCR (Fig. 1).

Immunofluorescence staining for $\alpha_v\beta_3$ shows specificity of the anti-$\alpha_v\beta_3$ antibody (MAB1979) (Fig. 2) used for immunohistochemical analysis of frozen tissue sections from sarcoma patients (Fig. 3).

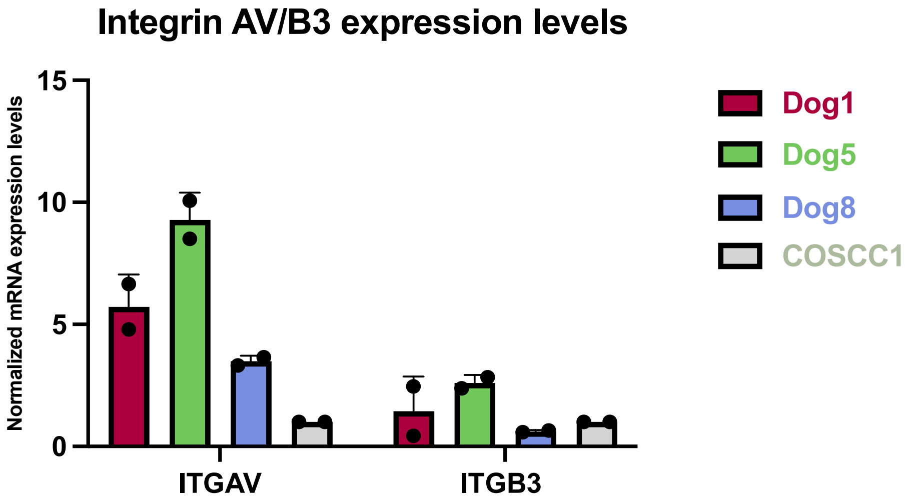

Fig. 1 Relative mRNA levels of integrin α_v (ITGAV) and integrin β_3 (ITGB3) in Dog1, Dog5 and Dog8, normalized to expression in epithelial COSCC1 cells.

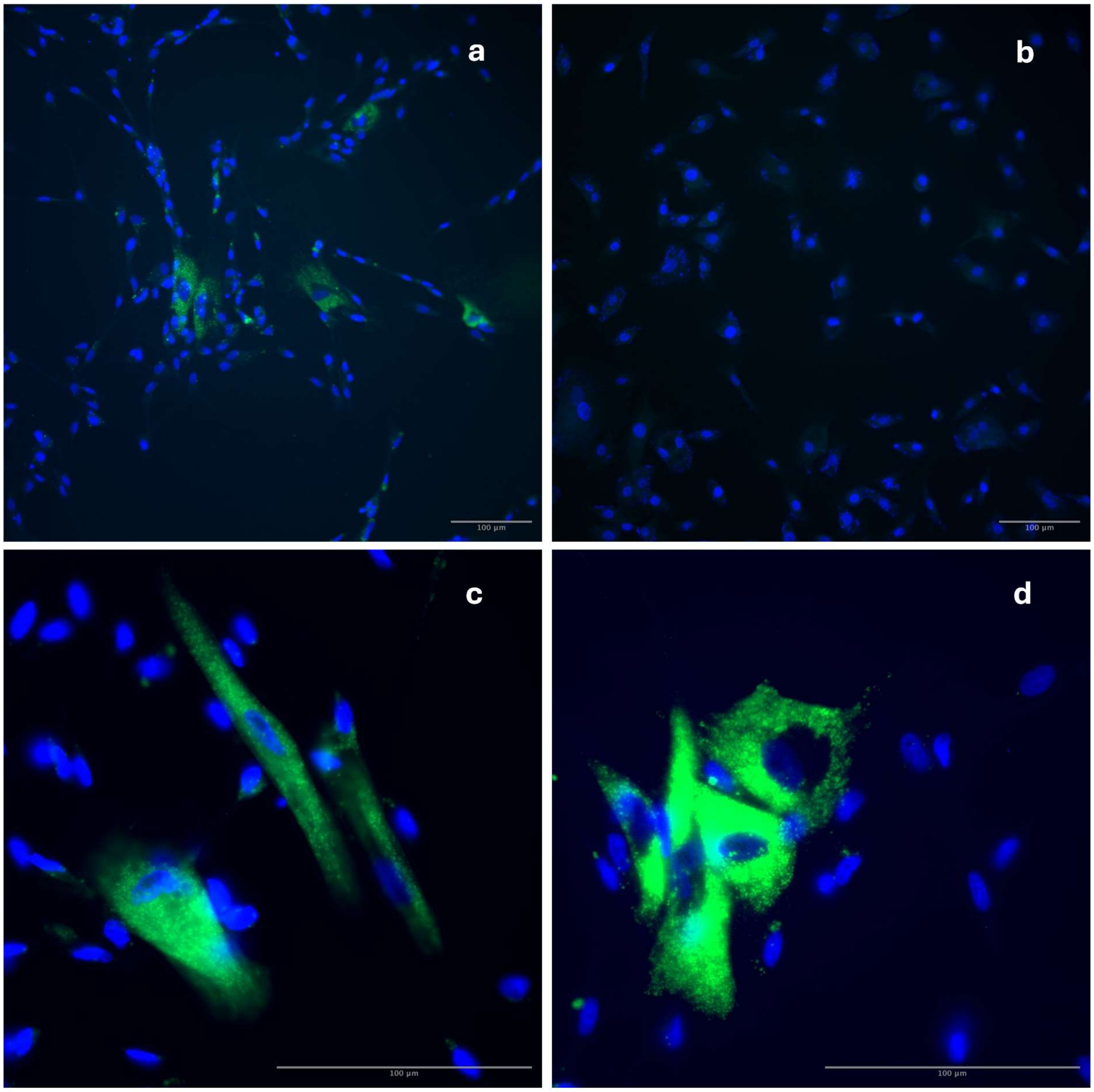

Fig. 2 Immunofluorescence staining of canine soft tissue sarcoma cells (STS) from Dog5 and COSCC1 cells treated with monoclonal anti-integrin $\alpha_v\beta_3$ antibody (clone LM609 (MAB1976)) and goat anti-mouse IgG (H+L) Alexa Fluor 488 (green). The nuclei were counterstained with DAPI (blue).

(a) In a subset of STS cells $\alpha_v\beta_3$ integrin was clearly labelled with MAB1976 (20x magnification). (b) COSSCC1 cells served as negative control lacking $\alpha_v\beta_3$ integrin expression. No detectable stain was observed for $\alpha_v\beta_3$ integrin, while nuclear antigens were labelled with DAPI. (c and d) show the $\alpha_v\beta_3$ labelled STS cell at a higher magnification (63x). Exposure time 1.98 seconds; 2x2binning; scale bar 100 μm.

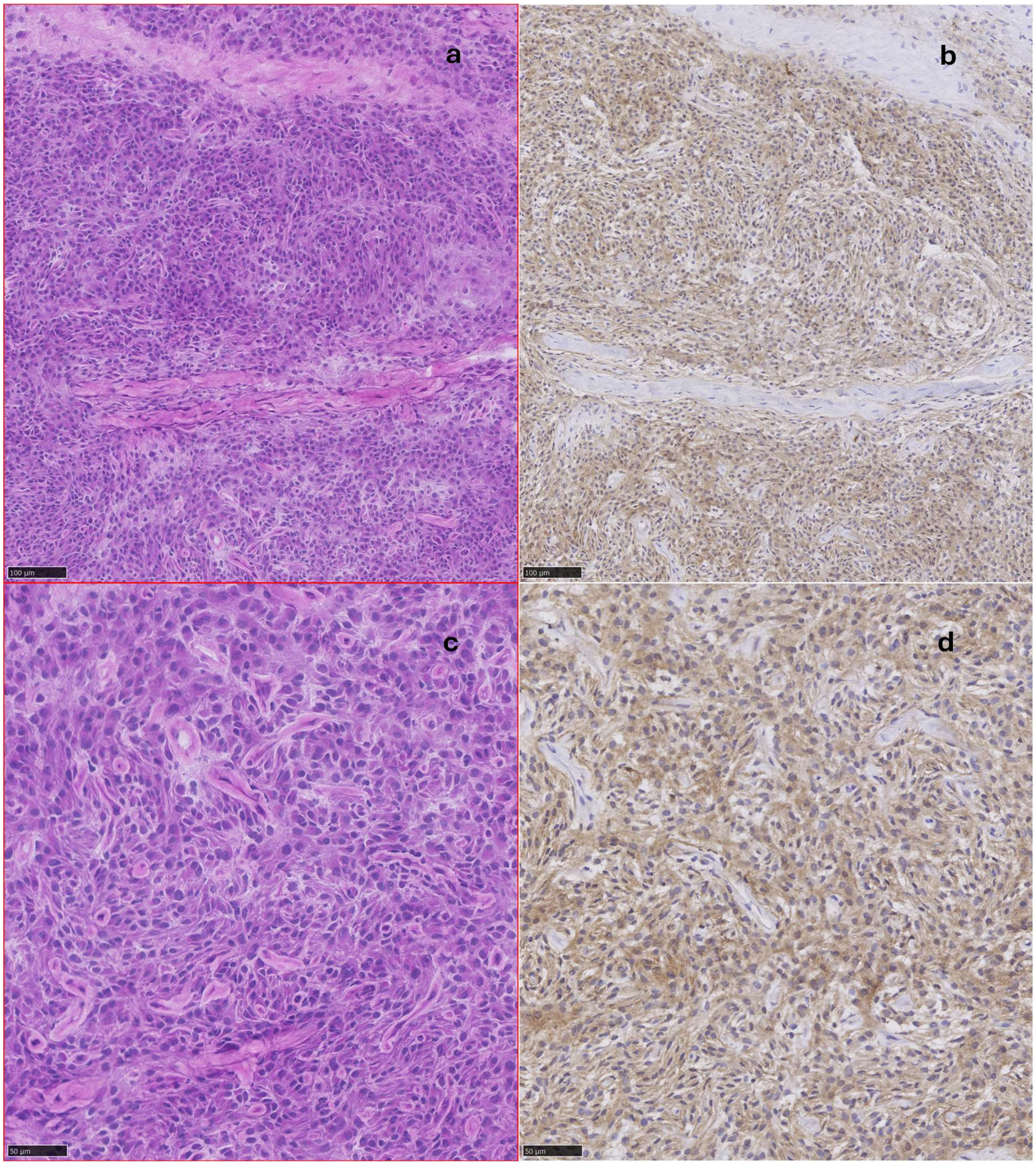

Fig. 3 Representative images of HE (a, c) and anti-$\alpha_v\beta_3$ integrin (b, d) (MAB1976) stained frozen tumor sections of DOG5. The tumor cells of this perivascular wall tumor sample show a strong positivity for $\alpha_v\beta_3$ integrin. (20x magnification a and b, scale bar 100 μm; 40x magnification c and d, scale bar 50 μm)

3. References

1. Beer P, Pauli C, Haberecker M, Grest P, Beebe E, Fuchs D, et al. Cross-species evaluation of fibroblast activation protein alpha as potential imaging target for soft tissue sarcoma: a comparative immunohistochemical study in humans, dogs, and cats. Front Oncol. 2023;13:1210004. doi:10.3389/fonc.2023.1210004.
2. Guscetti F, Nassiri S, Beebe E, Rito Brandao I, Graf R, Markkanen E. Molecular homology between canine spontaneous oral squamous cell carcinomas and human head-and-neck squamous cell carcinomas reveals disease drivers and therapeutic vulnerabilities. Neoplasia. 2020;22:778-88. doi:10.1016/j.neo.2020.10.003.
