# Supplementary material for: Evaluation of a targeted anti-αvβ3 integrin near-infrared fluorescent dye for fluorescence-guided resection of naturally occurring soft tissue sarcomas in dogs
Source: Eur J Nucl Med Mol Imaging. 2024 Oct 22;52(3):1137–48. doi: 10.1007/s00259-024-06953-x (PMC11754361; doi:10.1007/s00259-024-06953-x)
Supplement: Supplementary file 5 — Supplementary file5 (PDF 4410 KB) [file 259_2024_6953_MOESM5_ESM.pdf]

Supplementary Information (SI) 4

Near infrared fluorescent image analysis and histopathology

Evaluation of a targeted anti-$\alpha_v\beta_3$ integrin near-infrared fluorescent dye for fluorescence-guided resection of naturally occurring soft tissue sarcomas in dogs.

European Journal of Nuclear Medicine and Molecular Imaging

Patricia Beer[1*], Paula Grest[2], Christiane Krudewig[2], Chris Staudinger[3], Stefanie Ohlerth[3], Carla Rohrer Bley[4], Armin Jarosch[5], Houria Ech-Cherif[6], Enni Markkanen[6], Brian Park[1], Mirja Christine Nolff[1]

[1]Clinic for Small Animal Surgery, University Animal Hospital, Vetsuisse Faculty, University of Zurich, Zurich, Switzerland

[2]Institute of Veterinary Pathology, Vetsuisse Faculty, University of Zurich, Zurich, Switzerland

[3]Clinic for Diagnostic Imaging, University Animal Hospital, Vetsuisse Faculty Zurich, University Zurich, Zurich, Switzerland

[4]Division of Radiation Oncology, University Animal Hospital, Vetsuisse Faculty Zurich, University Zurich, Zurich, Switzerland

[5]Department of Pathology, Charité-Universitätsmedizin Berlin, Corporate Member of Freie Universität Berlin and Humboldt-Universität zu Berlin, Berlin, Germany

[6]Institute of Veterinary Pharmacology and Toxicology, Vetsuisse Faculty, University of Zurich, Zurich, Switzerland

*corresponding author: pbeer@vetclinics.uzh.ch

1. Materials and Methods

1.1. Preoperative NIRF imaging of the tumor

Imaging of the clipped tumor site was started prior to the injection of Angiostamp™ or placebo, and repeated immediately after injection, at 15 min, 30min, 45 min, 1 h, 2 h, 3 h, 4 h, 10 h, and every 4 h after until surgery using the VisionSense™ VS3 Iridium System (Medtronic, Minneapolis, USA). Imaging was performed under standardized ambient light conditions. Clinical examinations (heart frequency, respiratory rate, blood pressure, evaluation of mucous membranes, swelling and oedema) were performed to detect potential local or systemic side effects. Images were subjectively evaluated for the time until detection of the first NIRF signal visible in the tumor region, unspecific peritumoral signal, and tumor delineation after washout of background fluorescence.

1.2. Intraoperative transcutaneous NIRF imaging of the tumor in situ

Intraoperatively, prior to the first cut and prior to NIRF imaging, the tumor was palpated, visually assessed and marked on the skin (Fig. 1). A resection margin of 1 cm around and 3 cm around the tumor were marked. In dogs of the NIRF group, imaging was started, and the resection margins were adapted if required. Figure 4 shows the eight tumors of the NIRF group with transcutaneously detectable NIRF signal. In two dogs no tumor-specific signal was visible due to the deep localization of the PNST in the sciatic nerve.

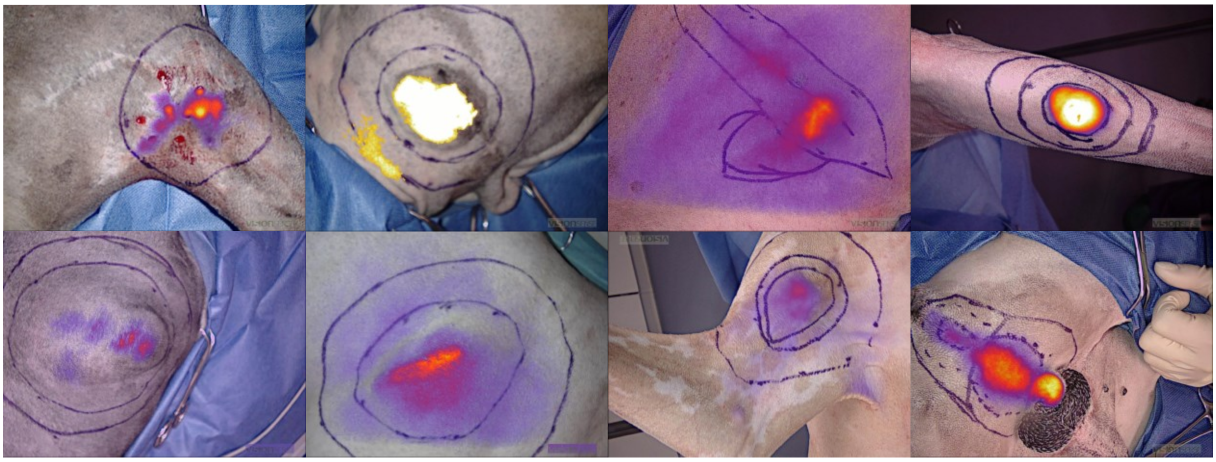

Fig. 1 Intraoperative NIRF colour fused images of eight tumors with a detectable transcutaneous fluorescent signal. (First row from left to right: dog 1, 4, 6, 7; Second row from left to right: dog 8, 11, 12, 18)

The NIRF white light and IR images of the tumors were analysed using ImageJ 1.53k (National Institutes of Health, Bethesda, MD, USA) after predefined regions of interest (ROI) were selected in the white light and IR images. The ROIs were transferred to the greyscale IR image (72 dpi, 24 bits per pixel (RGB)) to allow measurement of NIRF signal intensity in all ROIs to determine mean fluorescent

intensity (MFI), minimum, maximum, and standard deviation of the MFI measured in arbitrary units (Pixel) ranging from 0 to 256.

In the white light image, a standardized ROI (size 8277 Pixel) was set in a region of normal skin adjacent to the surgical site (background) (Fig. 2c) and in the centre of the tumor (ROI T) (d). Furthermore, the visible tumor was delineated to receive a ROI labelled visible T (e). The fluorescent area was encircled in the IR image (NIR T) (f) and was superimposed with the visible tumor ROI using ImageJs function AND to receive an overlapping area labelled overlay T (g). The 1 cm margin was delineated using the intraoperative marking in the white light image and the visible tumor ROI was subtracted from this area using the function XOR to obtain an area 1cm around the gross tumor (1 cm area) (h). The same was performed for the 3 cm margin, whereby the 1cm margin including the tumor area was subtracted (3 cm area) (i).

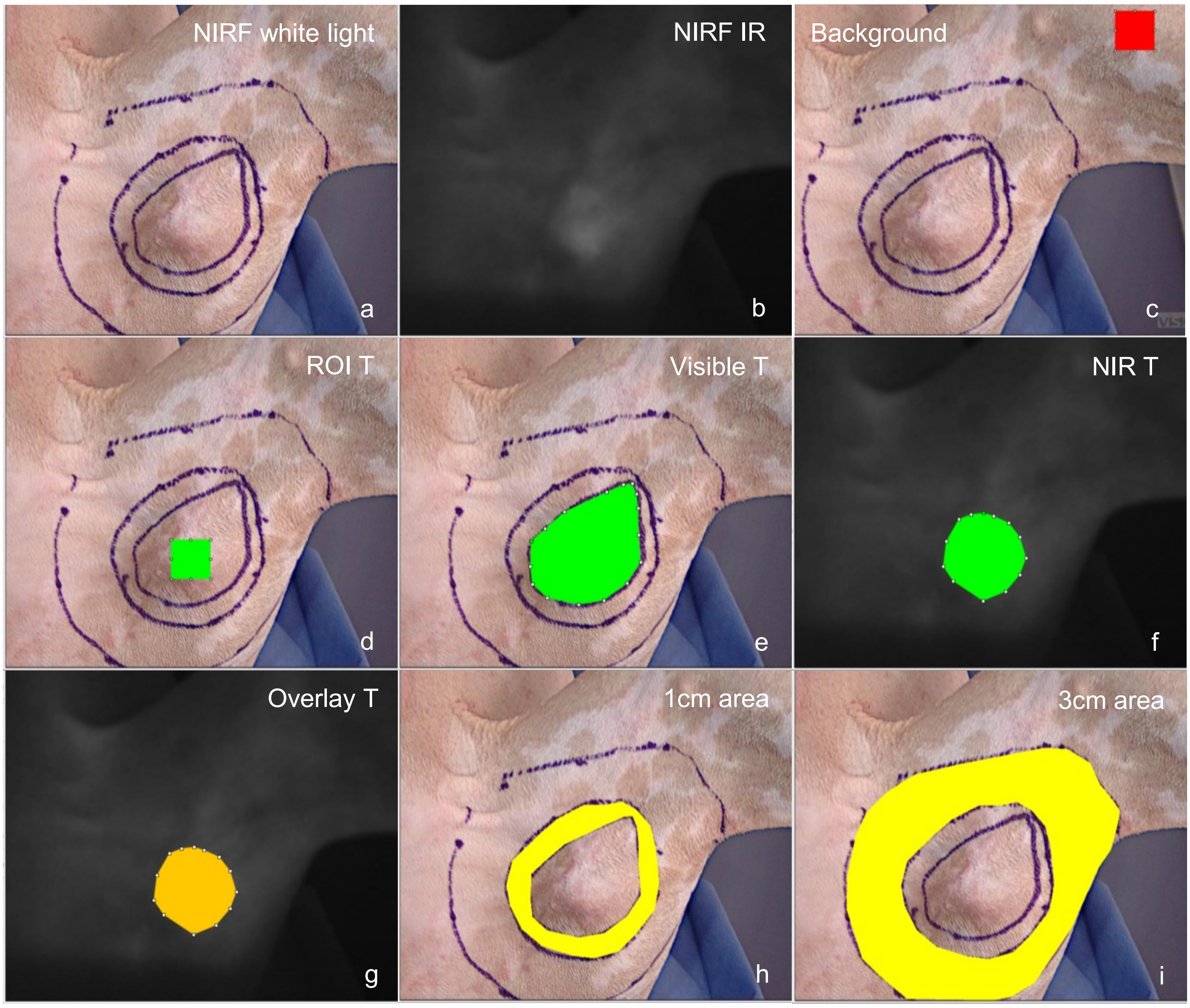

Fig. 2 Example for post-processing of intraoperative transcutaneous NIRF images of the tumor *in situ*. In the white light and IR tumor images predefined ROIs were selected and measurement of the fluorescent signal was performed using ImageJ 1.53k.

The tumor-to-background ratio (TBR) was calculated by dividing the ROI T by the background ROI. SBR were obtained by comparing the MFI of the background ROI with the visible T ROI, the 1 cm area ROI as well as the 3 cm area ROI and by comparing the different areas of the skin with each other. The SBR of all patients were then graphically compared with each other using a box plot.

The Dice coefficient (DC) was calculated to assess the similarity between the visible and palpable tumor and the transcutaneous visible NIRF signal using the formula: (2x MFI of overlay T)/(MFI of visible T + MFI of NIR T).

1.3. NIRF imaging of native tissue

Native tissue biopsies

Tissue biopsies of the tumor, the 1 cm margin, 3 cm margin and the tumor bed were taken using a 4 mm punch biopsy or a scalpel. The size of the samples was less then 1x1x1 cm and the number of samples taken per localization ranged between 1 to 3. In the two dogs suffering from a PNST of the sciatic nerve, a sample of the tumor, of the nerve in a transition zone between high signal and no signal assumed to be non-neoplastic and of the non-fluorescent nerve at the resection margin were taken. NIRF black table imaging was performed immediately after sampling. Overall, 10 images of tumor biopsies, 10 of 1 cm margin, 6 of the 3 cm margin and 10 tumor bed biopsies were available for analysis.

The NIRF white light and NIRF IR images were analyzed using ImageJ (Fig. 3a, b). A standardized ROI (size 8277 Pixel) was set in a corner of the white light image without tissue (background) (c) and the whole visible tissue was encircled (d). The ROIs were transferred to the corresponding IR image and the MFI was measured.

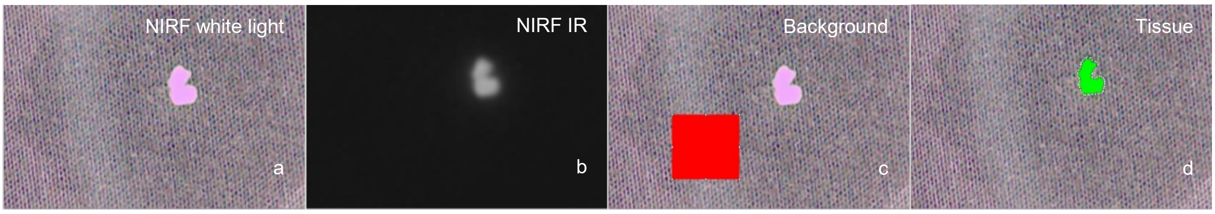

Fig. 3 Example of a biopsy taken from a PNST used for NIRF image analysis.

SBR were calculated by dividing the MFI of the tissue ROI by the MFI of the background ROI. SBR ratios of the different tissues (tumor, 1 cm, 3 cm margin and tumor bed) were then graphically displayed as box plots.

Native tumor specimen (skin turned towards the camera head)

NIRF image analysis of the native tumor specimen was performed as described for the transcutaneous imaging of the tumor, the background ROI was set in a region without visible tissue (Fig. 4c). Eight images were available for analysis. The Dice coefficient (DC) was calculated to assess the similarity between the visible and palpable tumor (visible T) and the visible NIRF signal (NIR T).

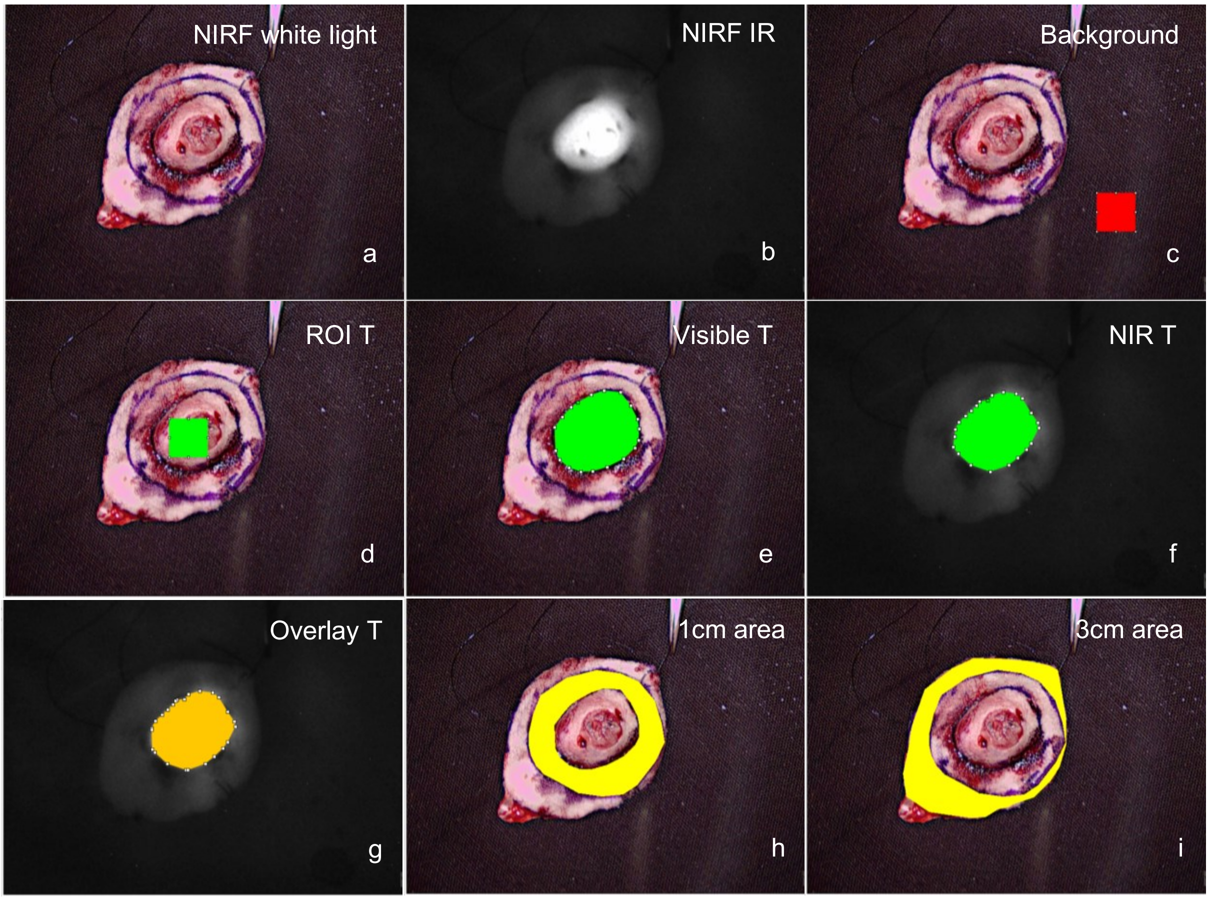

Fig. 4 Black table imaging and NIRF image analysis of the native tumor specimen with the side of the skin turned towards the camera head.

<u>Native tumor specimen (deep side of the specimen turned towards the camera head)</u>

The background ROI was set into a region without visible tissue in the white light image (background) (Fig. 5c) and the same standardized ROI was placed centrally in the region of the tumor (ROI T) (d). Seven images were available for analysis.

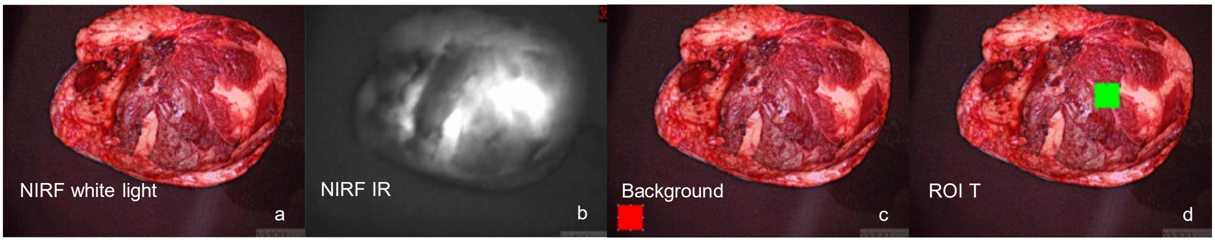

Fig. 5 Black table imaging and NIRF image analysis of the native tumor specimen with the deep side turned towards the camera head.

1.4. NIRF imaging of tumor bed

The images of the tumor bed (Fig. 6a, b) were acquired after the resection of the tumor and all additional fluorescent tissue considered suspicious for neoplasia. Six images were available for analysis.

The background ROI was placed in an area of skin adjacent to the tumor bed in the white light image (Fig. 6c) and the tumor bed was encircled (d). In cases with a high NIRF signal in the tumor bed, this area was marked as high signal ROI in the IR image (e). ROIs were again transferred to the IR image for MFI measurements (f).

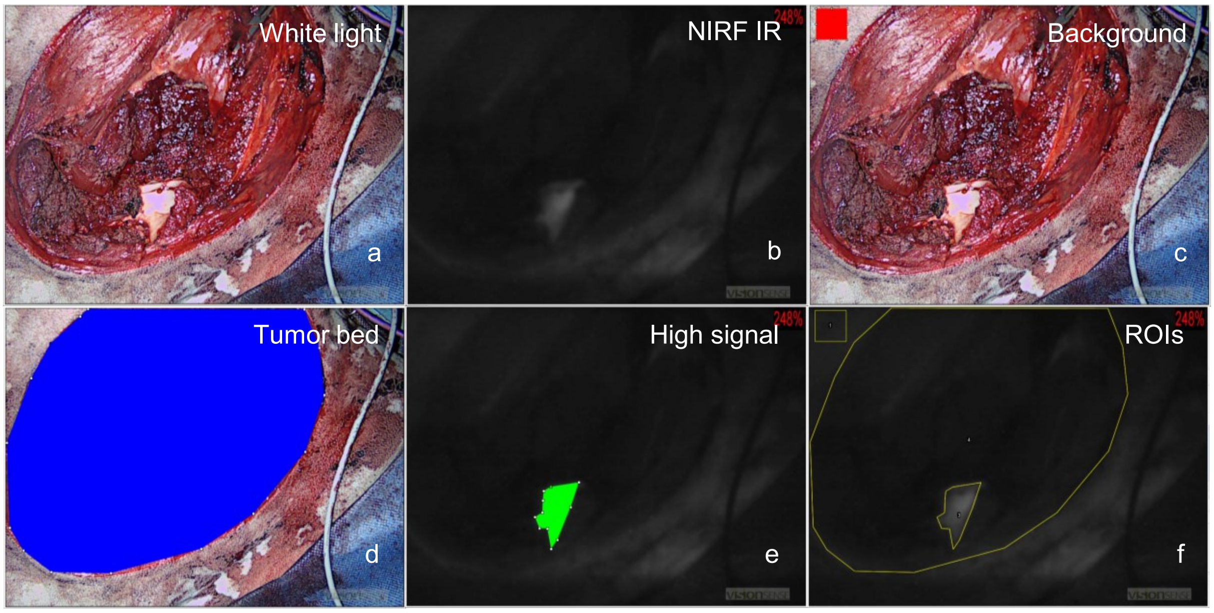

Fig. 6 Analysis of the fluorescent signal in the tumor bed after tumor resection using ImageJ. A high residual fluorescent signal was visible in this case due to dye accumulation in a piece of bone after partial scapulectomy.

The tumor bed to background ratio was measured by comparing the MFI of the entire tumor bed with the MFI of the background ROI. In addition, the SBR of areas with high residual fluorescence were assessed.

1.5. Tumor specimen fixation and trimming

After black table imaging the native tumor specimen was pinned on a crock plate and the edges were inked (Fig. 7a). The specimens were fixed in 10% formalin for 24 h-72 h depending on the size of the resected tissue. After fixation, the standard pathological examination started with a macroscopic assessment of the specimen, carried out by a board-certified veterinary pathologist (P.G.), a trained PhD student (P.B.) and/or a board-certified veterinary surgeon (M.N.). A cross-sectioning technique was used to measure the tumor size and distances of macroscopically tumor-free margins. Standard of care tissue sections were selected on the quarter sections for the histopathological assessment (b). The specimen was then bread loafed, and all sections with tumor or visible NIR signal were evaluated (c). All tissue

sections underwent black table imaging prior of being paraffin embedded (g). Paraffin blocks were then routinely cut, and tissue sections were HE stained and digitalized. A tumor diagnosis and grading were performed by a veterinarian pathologist and for tumors of the NIRF group a STS subtype classification was carried out by a human pathologist (A.J.) based on morphological and immunohistochemical characteristics as applied for human STS.

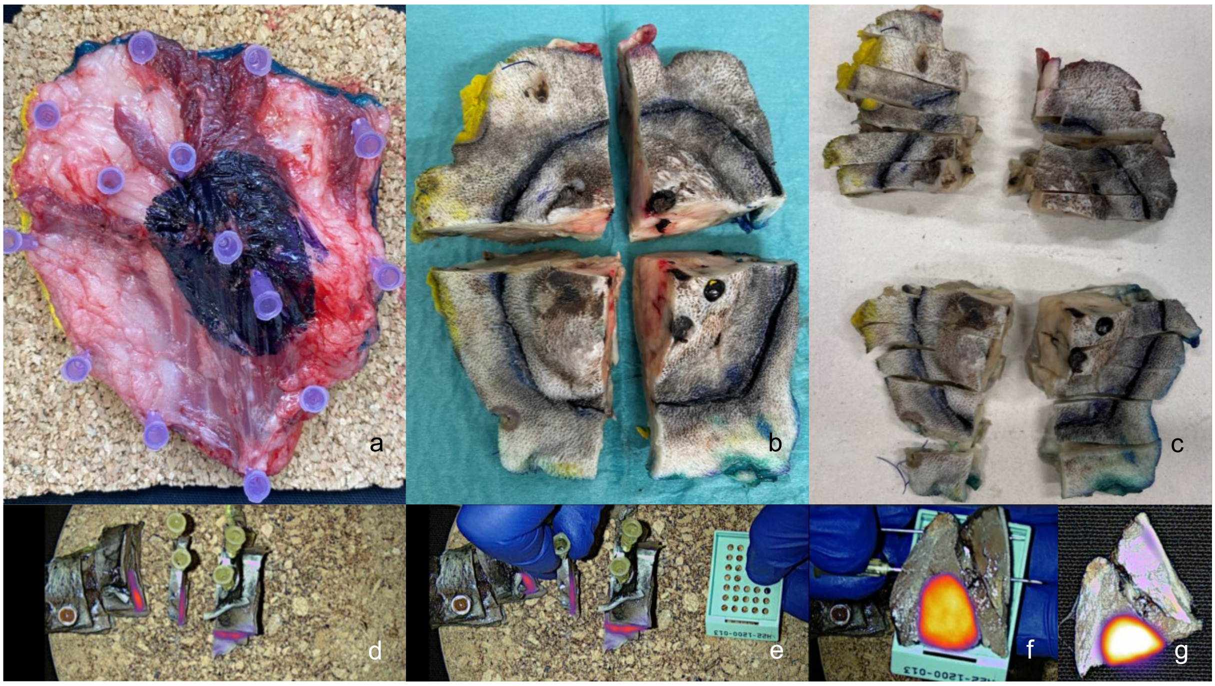

Fig. 7 Specimen trimming and selection of areas for paraffin embedding.

1.6. NIRF imaging of formalin fixated tissue

1.6.1. Subjective visual assessment of the NIRF signal and conformity with histology

NIRF images of the FFT sections of the tumor sections were used to subjectively assess the correspondence of a positive NIRF signal with histologically confirmed tumor extension in the corresponding histological images. An experienced veterinary pathologist (P.G.) and a trained PhD student (P.B.) reviewed all slides for the presence or absence of tumor tissue. Slides were classified as non-neoplastic or neoplastic. The corresponding IR images of the FFT slices were visually reviewed by P.B. for the presence or absence of a NIRF signal. Slices were classified as being NIRF positive with a visible NIRF signal, or negative without fluorescence visible. The results were used to determine the rate for true positive signal (NIRF signal and tumor present), true negative (no NIRF signal and no tumor), false positives (NIRF signal but no tumor) and false negatives (no NIRF signal but tumor).

1.6.2.Quantification of the NIRF signal and correlation with tumor area extent

Image registration

Image registration of the digitalized HE stained tissue section (300 dpi, 24 bits per pixel (RGB)) and the corresponding NIRF white light image of the FFT (72 dpi, 24 bits per pixel (RGB)) was performed using the software MATLAB R2023a (Mathworks Natick, Massachusetts, USA). Figure 8a shows the 2D image registration program and the images to be aligned. Distinct points at the outline of the tissue discernible in the NIRF white light image were manually selected (P.B.) and the corresponding points were marked in the histological slide. The points were then automatically aligned to receive a histological image shape matched with the NIRF visible image by applying a feature-based algorithm (b). The registered HE image (96 dpi, 24 bits per pixel (RGB)) was used for further analysis (c).

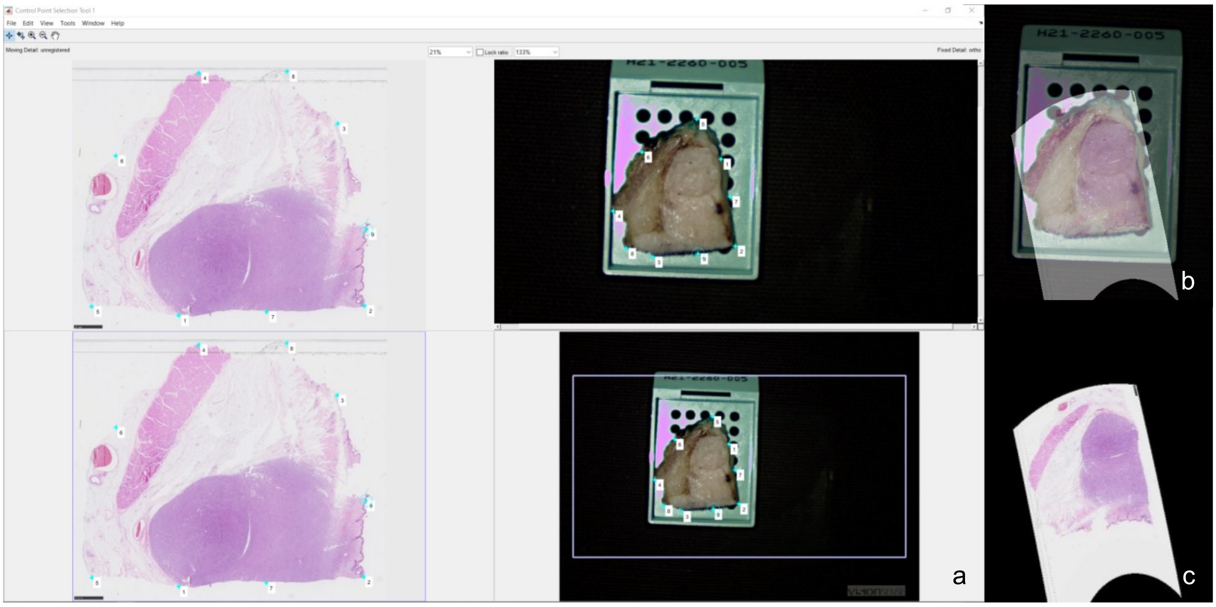

Fig. 8 2D image registration process using MATLAB R2023a (Mathworks Natick, Massachusetts, USA).

ROI selection and NIRF signal measurements

ROI selection and NIR signal intensity measurements were performed using the registered HE image (Fig. 9a), the white light image (b) and the IR image (c). A rectangular background ROI (size 8277 Pixel) was set into a corner of the white light image representing an area without tissue (background) (d). First, the grey scale IR image was used to encircle the area with a clearly visible NIRF signal (NIR T) (e). In the registered HE image the entire tissue was encircled and included in an ROI named HE tissue (f). Likewise, the histologically confirmed tumor tissue was delineated (HE T) (g). The peritumoral tissue in the histological section was computed by subtracting the ROI HE T from the ROI HE tissue (peritumoral HE) (function XOR) (h). The white light image was used to set a ROI around the entire visible tissue (visible tissue) (i). The HE T and visible tissue ROI were superimposed (function: AND)

to receive a ROI of visible tissue resembling the tumor area (visible T) (j). The visible T ROI was subtracted from visible tissue ROI using XOR to receive a peritumoral tissue ROI (visible peritumoral) (function XOR) (k). The ROIs HE T and NIR T were superimposed to select a ROI including only the overlapping area (overlay T) (l) using the function AND. Likewise, the ROI HE tissue and visible NIR were superimposed (overlay tissue) (m). By subtracting the NIR T ROI from the visible tissue ROI (function XOR) the region of peritumoral tissue in the NIR image was delineated (peritumoral NIR) (n). Measurements of the NIRF signal intensity of all ROIs was again performed in the IR image (o) as described above.

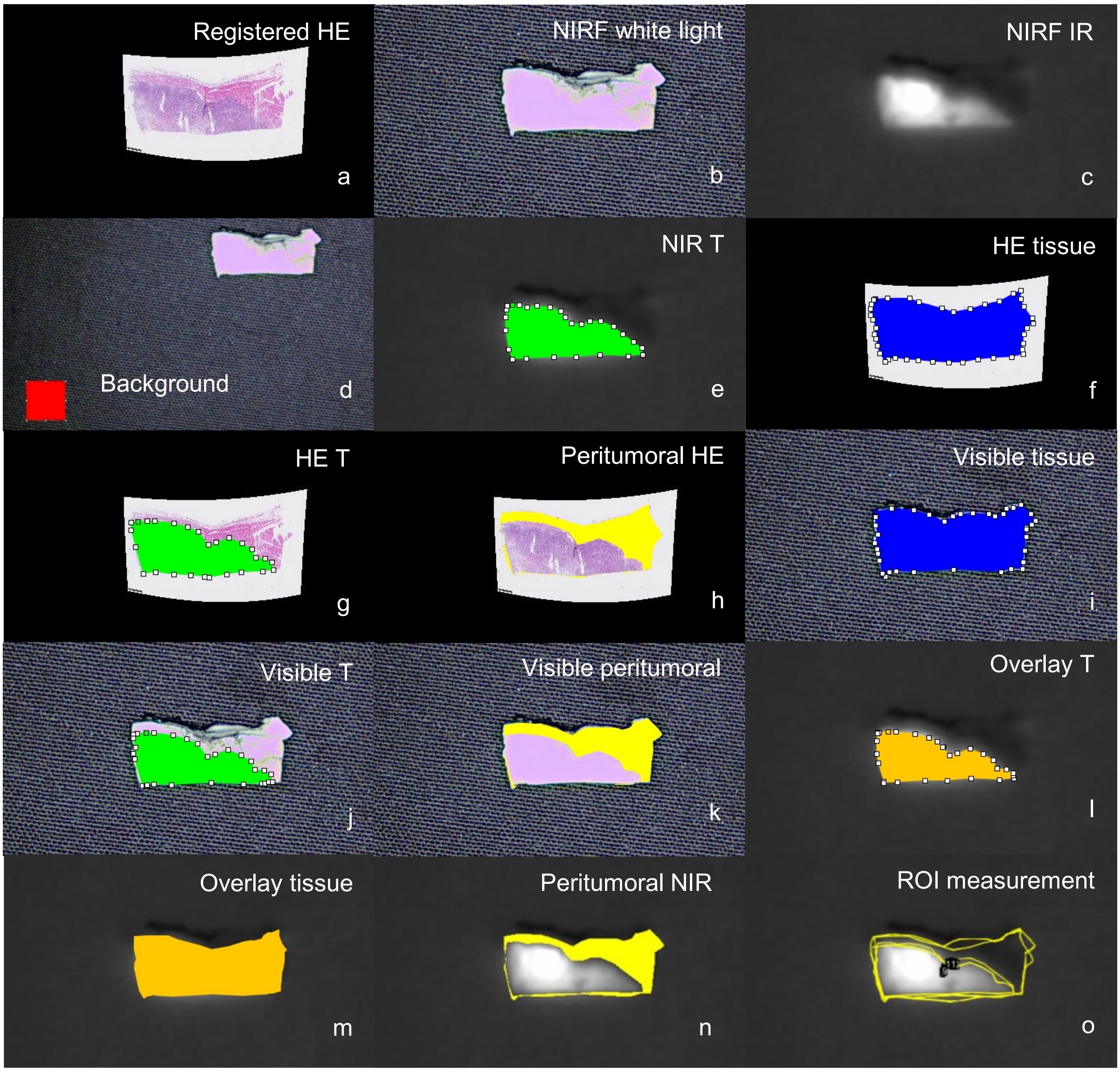

Fig. 9: Selection of regions of interest in NIRF images of formalin fixed tissue and the corresponding registered histological slide using ImageJ 1.53k.

<u>Calculation of signal ratios and Dice Coefficients</u>

In FFT IR images the TBR was calculated by dividing the MFI of the ROI resembling histologically confirmed tumor tissue (visible tumor) by the MFI of the background ROI. The tumor-to-peritumoral

tissue ratio (TPR) was defined as the MFI of the visible tumor ROI divided by the MFI of the visible peritumoral tissue ROI.

The DC is a similarity measurement, a ratio of the intersection of two areas over their union. It ranges between 0, no overlap, and 1, complete overlap and perfect agreement of the two areas. Figure 3 provides an exemplary illustration of the steps required to calculate the DC of tumor ROIs. The DC was used to assess if the area of histologically confirmed tumor (HE T) and the area of a high fluorescent signal (NIR T) are overlapping to a high degree meaning that NIRF imaging using Angiostamp^{TM} allows a precise visual tumor demarcation in FFT. Furthermore, the DC of the tissue ROIs, the HE tissue and visible tissue, was calculated to assess the agreement of the areas after image registration.

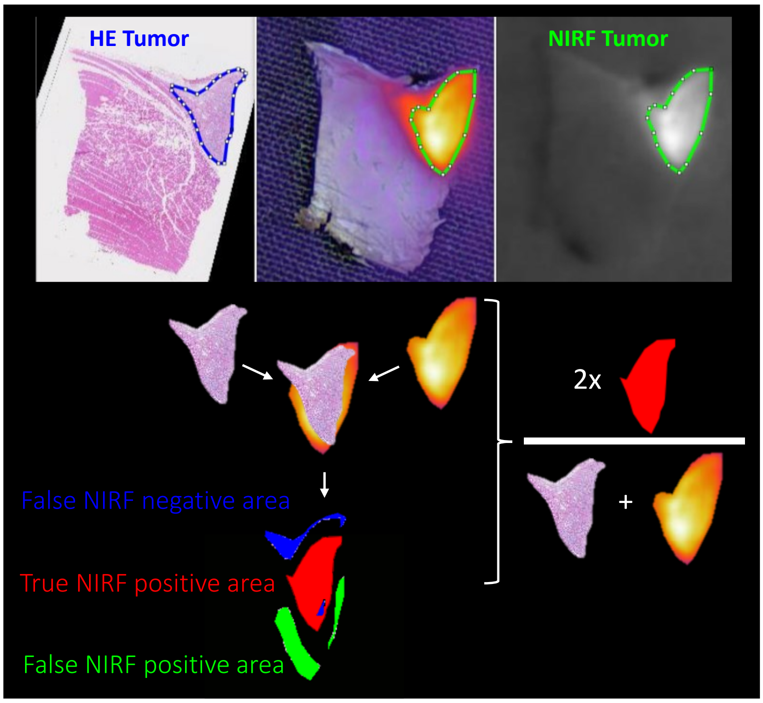

Fig. 10 Illustration of the Dice coefficient calculation in NIRF images of formalin fixed tissue and corresponding HE stained tissue sections. The histologically confirmed tumor tissue and the fluorescent area are encircled, and an overlapping area (in red) is calculated using ImageJ. The red area resembles the NIRF positive area that correctly identified neoplastic tissue. The DC is the fraction of two times the overlapping area and the sum of the HE tumor and NIR tumor area.

1.7. Recurrence-free survival

Recurrence-free survival (RFS) was defined as the time period from the date of surgical excision to the date of local recurrence or/and distant metastasis. Cause of death was classified as sarcoma-related or sarcoma-unrelated. Dogs that died sarcoma-unrelated, were lost to follow-up or were still alive at the last follow-up were censored. RFS rate was estimated according to the Kaplan–Meier method and survival functions of the NIRF and control group were compared using the log-rank test. $P<0.05$ was considered statistically significant.

2. Results

2.1. Patient-related information and histopathology

Table 1: Signalment, tumor characteristics and surgical treatment for the enrolled 20 dogs.

Case nr	Group: control (CG) NIRF (NIRF)	Breed	Sex: male (m) m castrated (mc) female (f) f castrated (fc)	Age at surgery (years)	Primary or recurrent tumor	Tumor localization	Surgical intervention and wound closure
1	NIRF	mixed breed	m	12	recurrence	hind limb	3 cm lateral margin 1 fascial plane/primary closure
2	CG	Havanese Dog	mc	14	primary	lateral thorax	3 cm lateral margin 1 fascial plane/primary closure
3	CG	Rhodesian Ridgeback	mc	9	recurrence	hind limb	amputation/primary closure
4	NIRF	French Bulldog	fc	10	primary	neck	3 cm lateral margin 1 fascial plane/primary closure
5	CG	mixed breed	mc	11	primary	lateral thorax	3 cm lateral margin 1 fascial plane/primary closure
6	NIRF	Malinois	fc	6	recurrence	lateral thorax	thoracic wall resection and reconstruction (mesh)
7	NIRF	Blanc Suisse	fc	5	primary	front limb	3 cm lateral margin 1 fascial plane/brachial axial pattern flap
8	NIRF	mixed breed	mc	11	primary	ischiatic tuberosity	3 cm lateral margin 1 fascial plane/caudal superficial epigastric axial pattern flap
9	CG	Labrador Retriever	mc	8	primary	lateral thorax	3 cm lateral margin 1 fascial plane/primary closure
10	CG	Siberian Husky	m	12	recurrence	thorax sternal	3 cm lateral margin 1 fascial plane/primary closure
11	NIRF	English Bulldog	m	1	primary	lateral thorax	3 cm lateral margin 1 fascial plane/primary closure
12	NIRF	Podengo Portugues	fc	6	primary	front limb	amputation/primary closure
13	CG	mixed breed	mc	14	primary	lateral thorax	3 cm lateral margin 1 fascial plane/primary closure
14	CG	Golden Retriever	mc	11	primary	ischiatic tuberosity	3 cm lateral margin 1 fascial plane/primary closure
15	NIRF	Giant Schnauzer	mc	8	primary	N. ischiadicus	Hemipelvectomy, amputation, hemilaminectomy
16	NIRF	West Highland White Terrier	mc	6	primary	N. ischiadicus	Hemipelvectomy, amputation, hemilaminectomy
17	CG	mixed breed	mc	14	recurrence	hind limb	3 cm lateral margin 1 fascial plane/cranial superficial epigastric flap
18	NIRF	French Bulldog	m	9	recurrence	perineal/ scrotal	3 cm lateral margin 1 fascial plane/primary closure
19	CG	Gordon Setter	m	8	recurrence	thorax sternal	4 cm lateral margin 1 fascial plane
20	CG	mixed breed	fc	12	primary	thorax sternal	3 cm lateral margin 1 fascial plane/primary closure

Table 2: Follow-up and histopathological and immunohistochemical findings for the enrolled 20 dogs.

Case nr	Group: control (CG) NIRF (NIRF)	time between injection and imaging (h:min)	Recurrence (yes/no) and time to recurrence (days)	Alive or dead (reason for death)	Follow up (days)	Tumor entity PWT=1 STS NOS=2 cFS=3 PNST=4	Tumor grade	1 cm lateral margin complete or incomplete	completeness of resection (R0-R2)	$\alpha_v\beta_3$ integrin expression score
1	NIRF	36:10	no	dead (haemangio-sarcoma)	122	PWT	low	not available	R0	high
2	CG	36:10	no	alive	1028	PWT*	intermediate	not available	R0	high
3	CG	36:00	no	dead (gastritis, pancreatitis)	16	PWT*	high	complete	R0	high
4	NIRF	06:25	no	dead (aspiration pneumonia)	1	STS NOS	high	complete	R0	intermediate
5	CG	11:00	no	dead (suspected renal tumor)	696	PWT	low	complete	R0	high
6	NIRF	12:45	no	alive	875	STS NOS*	99999	complete	R0	high
7	NIRF	12:22	no	alive	868	PWT	low	complete	R0	high
8	NIRF	12:05	no	alive	819	PWT	low	complete	R0	high
9	CG	12:10	no	alive	805	FS	low	complete	R0	intermediate
10	CG	12:30	no	alive	791	PWT	intermediate	incomplete	R1	high
11	NIRF	12:10	no	alive	784	STS NOS	high	incomplete	R0	intermediate
12	NIRF	11:50	yes: 55	dead (metastases)	141	PWT	intermediate	incomplete	R1	low
13	CG	12:15	no	alive	681	PWT	low	complete	R0	high
14	CG	12:00	no	alive	651	PWT	low	complete	R0	high
15	NIRF	12:05	no	alive	557	PNST	99999	complete	R0	intermediate
16	NIRF	12:40	no	dead (melanoma, lung metastasis)	562	PNST	99999	complete	R0	high
17	CG	12:21	yes: 291	alive	552	PWT	low	incomplete	R1	high
18	NIRF	12:35	no	alive	511	PWT	low	incomplete	R0	high
19	CG	12:05	no	alive	462	PWT	intermediate	complete	R0	not available
20	CG	11:40	no	alive	385	PWT	low	complete	R0	high

PWT, perivascular wall tumor; STS NOS, soft tissue sarcoma not otherwise specified; cFS, canine fibrosarcoma; PNST, peripheral nerve sheath tumor

Table 3: Classification of postoperative complications diagnosed in 11 out of 20 dogs (55%) after Clavien-Dindo [1]. No pre- and intraoperative complications occurred.

**Case nr**	**Group: control (CG) NIRF (NIRF)**	**Postoperative complication: grade I grade II grade IIIa grade IIIb grade IV grade V**	**Type of complication**
1	NIRF	none	
2	CG	none	
3	CG	grade V	pancreatitis/ gastritis/oesophagitis/ death
4	NIRF	grade V	aspiration pneumonia/ death
5	CG	none	
6	NIRF	none	
7	NIRF	grade IIIb	partial flap necrosis/ deep SSI
8	NIRF	grade IIIb	partial flap necrosis/ seroma formation
9	CG	none	
10	CG	grade I	seroma
11	NIRF	none	
12	NIRF	grade I	seroma/ regurgitation/ vomiting
13	CG	none	
14	CG	grade I	seroma/ superficial SSI
15	NIRF	grade IIIb	deep SSI, L7-S1 subluxation/ instability after hemilaminectomy
16	NIRF	grade IIIb	deep SSI
17	CG	grade IIIb	partial flap necrosis
18	NIRF	none	
19	CG	grade IIIb	partial flap necrosis
20	CG	none	

2.1.1.Recurrence-free survival

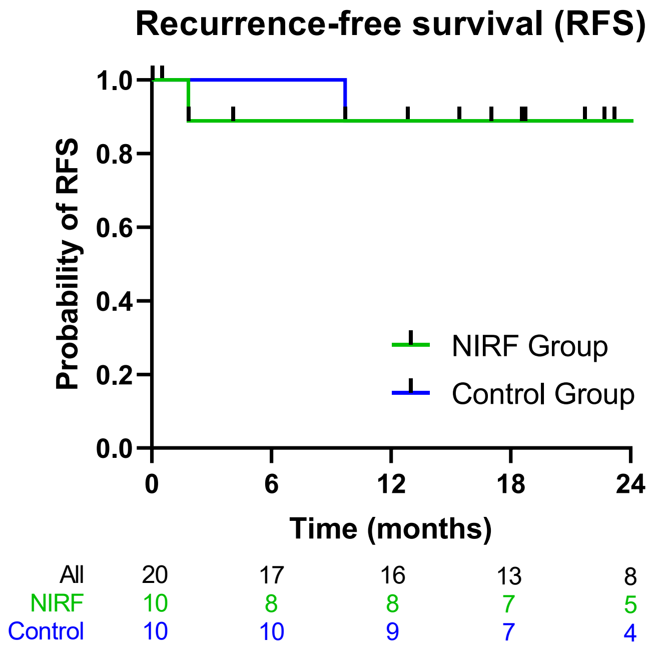

Fig. 11 Estimated Kaplan–Meier function of recurrence-free survival (RFS) for dogs undergoing surgical excision of STS stratified by the treatment group and number of patients at risk. Censored dogs are indicated by a thick mark. No differences in the RFS functions were detected between groups (p=0.929). Median recurrence-free time was not reached.

2.1.2. Comparison of veterinary and human STS subtype classification

Based on micromorphological characteristics and immunohistochemical analyses, the most common STS subtype using the veterinary medicine classification were PWT (n=5), which fits to the reported frequency of this tumor histotype [4-6]. In this study the four low-grade PWT corresponded to intermediate-grade subtypes of the human classification system [7] namely dermatofibrosarcoma protuberans (DFSP) (n=3) and inflammatory myofibroblastic tumors (IMT) (n=1). DFSP are described as being the most common dermal sarcoma type in humans [8]. IMT are ultra-rare tumors with the localization in the skin being even more rare then in other organs [9]. In humans DFSP and IMT rarely metastasize, but they tend to local recurrence in up to and over one third of patients [8-12]. None of the patients with a PWT grade 1 and diagnosed DFSP developed local recurrence or distant metastasis after complete resection in this study. Patient 18, a middle-aged male intact French Bulldog, that was affected by IMT was presented with the third local recurrence of this tumor in the perineal and scrotal region without signs of distant metastasis. After R0 resection the tumor did not recur so far. In this study, one PWT was classified as intermediate grade. The corresponding human diagnosis was a high-grade STS, most likely a spindle cell rhabdomyosarcoma (RMS), but molecular testing would have been required to secure molecular pathological diagnosis. Fitting to this aggressive tumor type in humans, especially if it occurs in adults [13], this dog developed local recurrence, metastasis and died sarcoma-related.

Secund most common diagnosis in dogs of this study were STS that could not be further specified based on routine diagnostics (STS NOS), but were judged as aggressive spindle cell neoplasms. Based on human STS criteria those tumors were classified as DFSP with fibrosarcomatous differentiation (FS-DFSP), undifferentiated pleomorphic sarcoma (UPS) and sclerosing epithelioid fibrosarcoma (SEF). Fibrosarcomatous transformation of DFSP (FS-DFSP) is rare and has a worse prognosis [8], with a higher rate of local recurrence and a higher metastatic potential [12, 14]. This corresponds to our clinical findings in patient 4 that had diagnosis of a STS NOS with lymph node metastasis. As this dog died due to aspiration pneumonia in the perioperative period, we could not perform a long-term follow-up after complete surgical tumor resection. Likewise, SEF is associated with a high rate of local recurrence and metastasis. Although it is a histologically low-grade tumor, SEF tend to be clinically high-grade [15]. The dog noted with the human diagnosis SEF received the veterinary diagnosis of a STS NOS.

Another tumor could not be further specified and was therefore also not graded despite appearing morphologically aggressive. This dog was presented with a local recurrence and a concurrent lung nodule that could be confirmed as being a sarcoma metastasis. The corresponding human diagnosis was an UPS [16]. Two canine PNST were noted to have a similar histomorphological appearance to human MPNST.

Table 8: Comparative veterinary and human medicine STS subtype classification based on morphological characteristics and immunohistochemical analysis.

Patient	Pathological diagnosis		Tumor grade	Performed immunohistochemical analysis for human subtype classification
	Veterinary	Human	(veterinary)	
1	PWT	DFSP	Grade 1	CD34 (positive), SMA (negative), ki67(<1%), Pan-cytokeratin (negative), S100 (negative), Vimentin (positive), CD31 (negative), Desmin (negative), MDM2 (negative)
4	STS NOS	FS-DFSP	Grade 3	CD34 (positive), SMA (negative), ki67(average 1%; hot spots: until 10 %), Pan-cytokeratin (negative), S100 (negative), Vimentin (positive), CD31 (negative), Desmin (negative), MDM2 (negative).
6	STS NOS	UPS	ungraded	CD34 (negative, focal weak positive), SMA (negative, focal positive), ki67 (25%), Pan-cytokeratin (negative), S100 (negative), Vimentin (positive), CD31 (negative), Desmin (negative), MDM2 (negative), INI1 (positive).
7	PWT	DFSP	Grade 1	CD34 (positive), SMA (negative), ki67 (<1%), Pan-cytokeratin (negative), S100 (negative), Vimentin (positive), CD31 (negative), Desmin (negative), MDM2 (negative).
8	PWT	DFSP	Grade 1	CD34 (positive), SMA (negative), ki67(<1%), Pan-cytokeratin (negative), S100 (negative), Vimentin (positive), CD31 (negative), Desmin (negative), MDM2 (negative).
11	STS NOS	SEF	Grade 3	CD34 (negative), SMA (negative), ki67(average 1%; hot spots: until 5 %), Pan-cytokeratin (negative), S100 (negative), Vimentin (positive), CD31 (negative), Desmin (negative), MDM2 (negative), MUC4 (negative), INI1 positive.
12	PWT	Spindle cell RMS	Grade 2	CD34 (positive), Desmin (positive), SMA (negative), ki67: average 15%, MyoD1 (negative), Myogenin (Myf4) negative, CD34 (positive), S100 (negative), Vimentin (positive), CD31 (negative), Pan-cytokeratin (negative), MDM2 (negative).
15	PNST	MPNST	ungraded	S100 (negative), ki67 (average 20%), CD34 (negative), CD31 (negative), SMA (negative), Desmin (negative), MDM2 (negative), Vimentin (positive), Pan-cytokeratin (negative), MDM2 (negative).
16	PNST	MPNST	ungraded	S100 (focal weak positive), ki67 (average 20%), CD34 (negative), CD31 (negative), SMA (negative), Desmin (negative), MDM2 (negative), Vimentin (positive), Pan-cytokeratin (negative), MDM2 (negative).
18	PWT	IMT	Grade 1	CD34 (positive, weak to moderate), SMA (negative, focal weak positive), ki67 (5%), Pan-cytokeratin (negative), S100 (negative), Vimentin (positive), CD31 (negative), Desmin (negative), MDM2 (negative), ALK (positive).

PWT, perivascular wall tumor; STS NOS, soft tissue sarcoma not otherwise specified; PNST, peripheral nerve sheath tumor; DFSP, dermatofibrosarcoma protuberans (DFSP); FS-DFSP, DFSP with fibrosarcomatous differentiation; UPS, undifferentiated pleomorphic sarcoma; SEF, sclerosing epithelioid fibrosarcoma; RMS, spindle cell rhabdomyosarcoma (RMS); MPNST, malignant peripheral nerve sheath tumor; IMT, inflammatory myofibroblastic tumor

2.2. Preoperative near-infrared fluorescent imaging (NIRF) imaging of the tumor

Considerations must be given to the influence of the dye dosage and the time between injection and surgery on the NIRF imaging results. Wenk et al. used a dose of 0.3 mg/kg in cats [2] while in dogs Mery et al. injected 0.15 mg/kg the day before surgery [3]. We observed sufficient fluorescent signal and contrast using 0.15 mg/kg Angiostamp^{TM}. Our first patient was operated 36 h post injection, as this was the time interval to receive the best SBR determined in cats [2]. The secund patient of the NIRF group received Angiostamp^{TM} 6h prior to surgery. As in the first dog already 12 h after injection an adequate tumor contrast could be observed and in the 2nd patient a considerable background signal was still visible, all further patients underwent surgery 12 h post injection. For clinical practicability we suggest a time interval between injection and surgery of 12-16 h.

Table 4: Findings during preoperative NIRF tumor imaging *in situ*.

**Case nr.**	**Time point of first NIRF-signal in tumor region**	**Tumor well delineated transcutaneous**	**Further unspecific signal**
1	signal after 30 min	yes	no
4	low signal immediately post injection, increased within first 15 min	not immediately, but later yes	low signal in parts of surrounding skin
6	immediately	no	surrounding skin
7	immediately	yes	low signal in parts of skin
8	immediately, but well delineated after 15 min	no only parts of the tumor (skin pigmentation)	no
11	signal immediately after injection, but high background signal	no, only parts of the scar	surrounding skin
12	signal immediately after injection visible in the tumor but high background signal	yes	surrounding skin
15	no signal	no	no
16	no signal	no	surrounding skin
18	immediately	yes	perianal and perineal

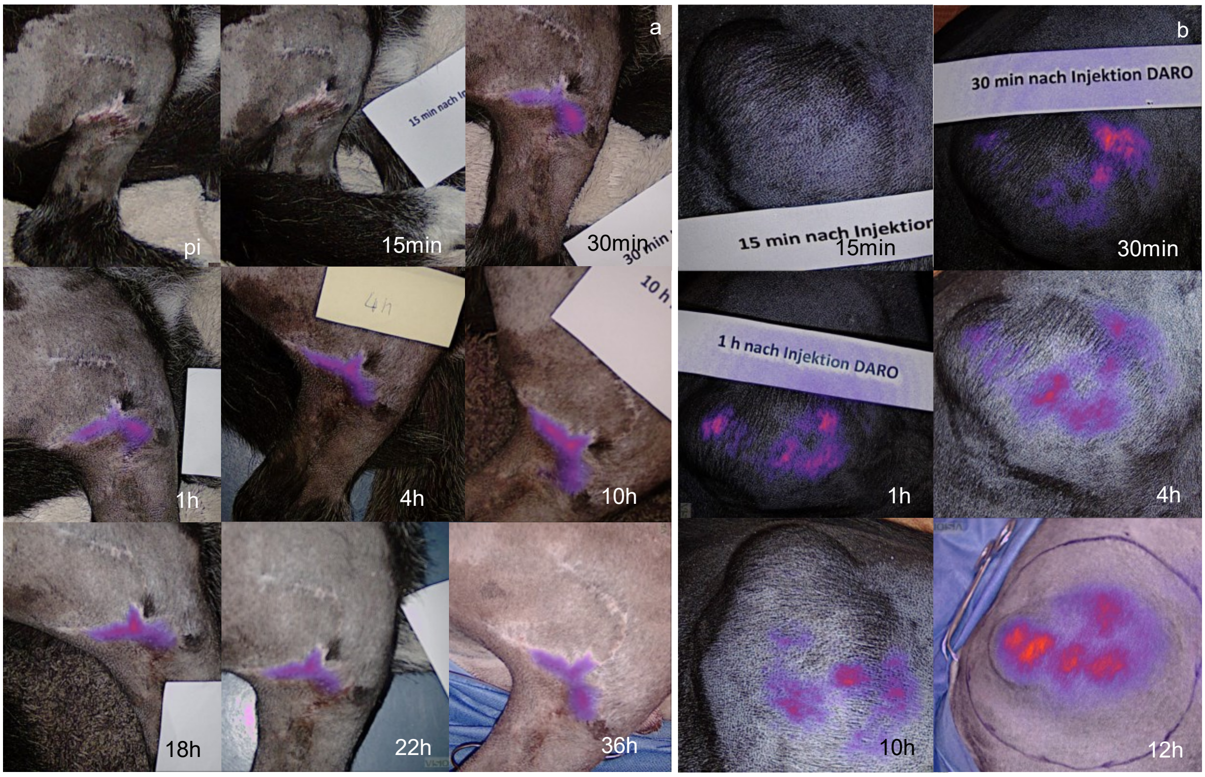

Fig. 12 Examples of transcutaneous tumor imaging post Angiostamp^{TM} injection (pi) and prior to surgery in two dogs. (a) Dog 1 was presented with a recurrent STS in the region of the right lateral hind limb. After 30 min a clear NIRF-signal was visible in the region of the scar in which a macroscopic tumor recurrence could be palpated and was visible on the CT scan. The signal extended more caudally towards the popliteal fossa in a region where histology later confirmed microscopic disease. The remaining scar on the lateral thigh was not accumulating fluorescent dye. The most distinct NIRF-signal within the tumor was visible after 10 to 18 h post injection. Surgery was performed 36 h pi. (b) Dog 8 was presented with a large primary STS in the region of the right ischiatic tuberosity extending cranially to the right flank. The first weak signal was visible within the first 15 min pi but was negatively affected by the pigmented skin. The transcutaneous signal remained patchy until surgery 12 h pi.

2.3. Subjective correlation of NIRF signal and true tumor extent

Table 5: Subjective visual assessment of the FFT formalin fixed tissue (FFT) sections of the trimmed tumor specimen and correspondence of a positive NIRF-signal with histologically confirmed tumor tissue in the corresponding histological images.

**Histology and NIRF imaging results**	**Number (%)**
histologically evaluated HE section in 20 dogs	356
histologically evaluated HE section in 10 dogs (NIRF group) with NIRF images of corresponding FFT available	148
NIRF-signal corresponds with histology (true positive or negative signal)	132/148 (89.2%)
Proportion of samples with tumor tissue	76/132 (57.6%)
Proportion of samples without tumor tissue	56/132 (42.4%)
NIRF-signal does not correspond with histology (false positive or negative signal)	16/148 (10.8%)
No NIRF-signal in neoplastic tissue	0/16 (0%)
Positive NIRF-signal in non-neoplastic tissue	16/16 (100%)
Positive NIRF-signal in lmpyh nodes, testicle, bone, circumanal gland	10/16 (62.5%)
Positive NIRF-signal in perituomoral non-neoplastic tissue	6/16 (37.5%)
Proportion of sections with a false positive NIRF-signal of all evaluated sections excluding sections of the false positive structures (lymph nodes, testicle, bone, circumanal gland)	6/138 (4.3%)

In 10.8% of the FFT samples (16/148) a NIRF-signal was visible while the tissue section was free from tumor tissue. Ten of those cases encompassed tissue of lymph nodes (n=6), bone (n=2), testicle (n=1) or circumanal gland (n=1), and 6 encompassed peritumoral tissue. Within the peritumoral tissue reactive inflammatory changes were observed that corresponded to the localization of the NIRF-signal in five cases (cystic lesion, granulation tissue, reactive muscle fibres, subcutaneous peritumoral inflammatory infiltration). In one sample no histopathological changes were observed. Although two fluorescent lymph nodes were metastatic (carcinoma, sarcoma metastasis) the fluorescent signal was diffuse and not localized in the single metastatic lesion as diagnosed histologically. All other false positive tissues did show a diffuse and strong signal with lacking evidence for pathohistological changes.

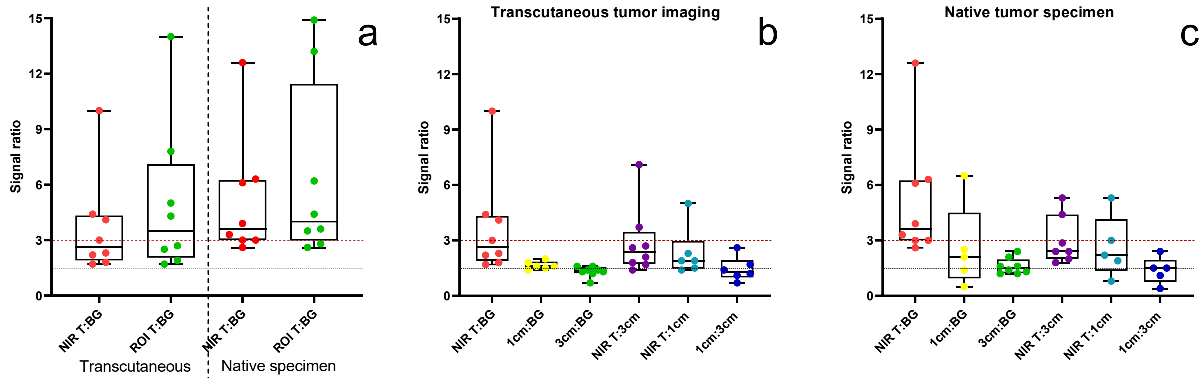

Fig. 13 We did not observe a difference in the tumor-to-background measurements of the transcutanous tumor images and the native tumor specimen images if using a standardized ROI placed within the tumor area (ROI T) or using a ROI encircling the whole fluorescent tumor (NIR T) ($p>0.9999$) (a) with a mean TBR of over 3 for all measurement methods (absolute values Table 7). Thus, we decided to use the ROI T:BG ratio as TBR for publication. Comparing the SBR of the different areas (area of the tumor, 1 cm margin, and 3 cm margin) in the transcutaneous images and the images of the native tumor specimen, a higher SBR was measured for the tumor compared to the 3 cm margin areas ($p<0.0016$). Overall, the signal ratios in these box plots illustrate that the NIRF-signal was the highest in the tumor and is decreasing towards the 1 cm margin area, the 3 cm margin area and the background.

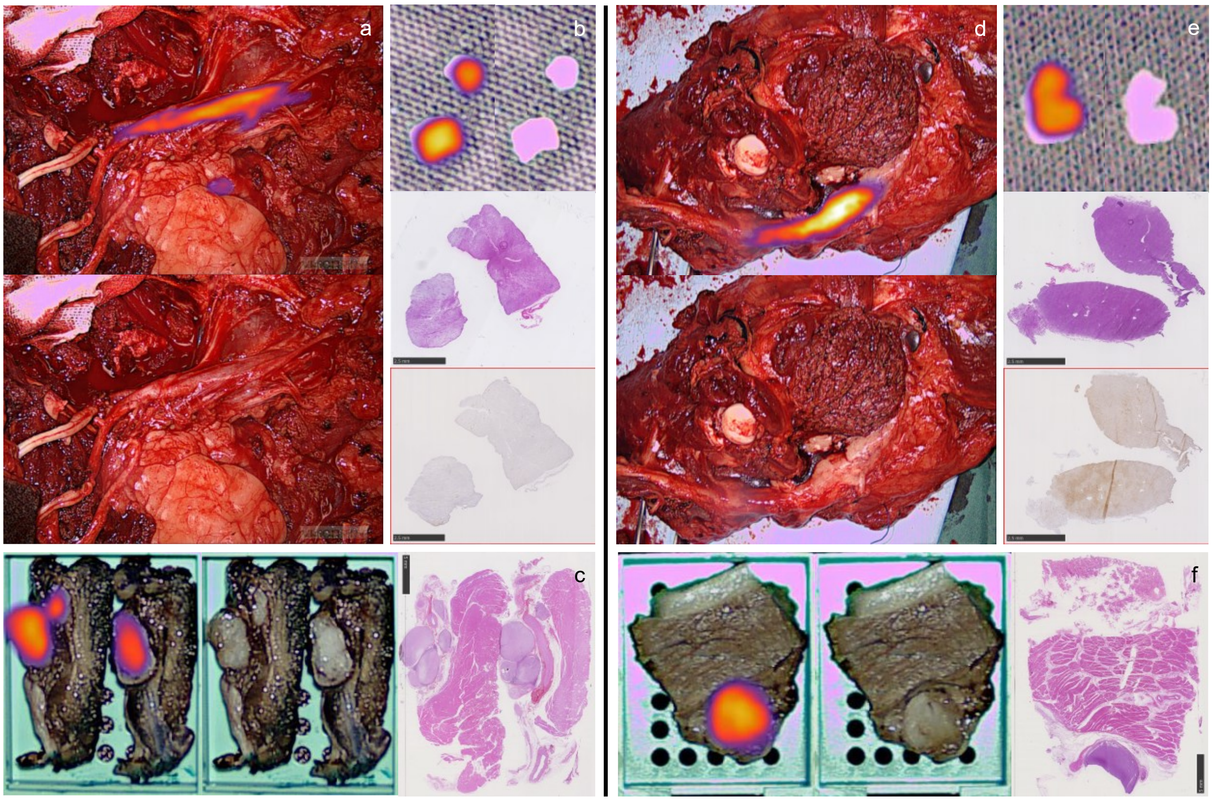

Fig. 14 NIRF imaging of peripheral nerve sheath tumors (PNST) of the sciatic nerve in dog 15 (a-c) and dog 16 (d-f). The NIRF-signal in the tumor enabled an intraoperative (a) and postoperative (d) tumor delineation. Biopsies taken from the tumor (b and e) showed a strong fluorescent signal and the presence of tumor tissue was confirmed by histology. $\alpha_v\beta_3$ integrin expression in the PNST was high in dog 16 (e) while the expression was lower in dog 15 (b). NIRF images of formalin fixed tumor and peritumoral tissue and the corresponding histological slides confirmed the correct localization of the NIRF-signal in areas of neoplasia (c and f).

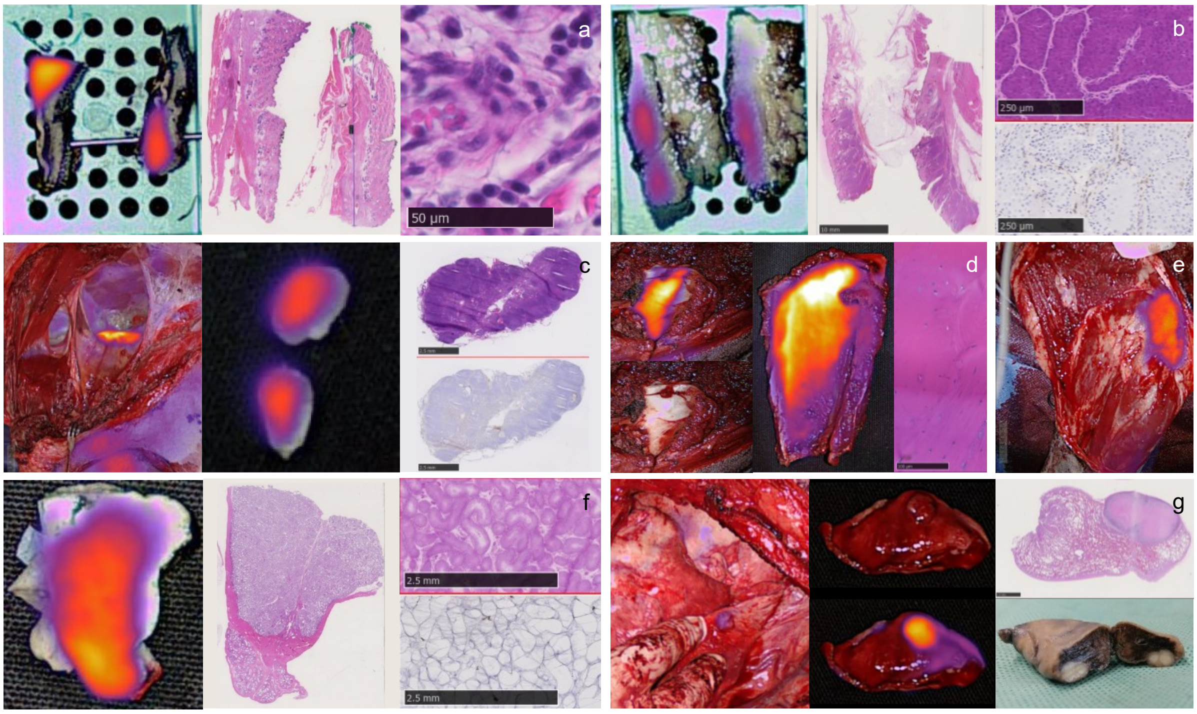

Fig. 15 Examples of fluorescent tissue structures. (a) shows a formalin fixed tissue (FFT) sample with a high fluorescent signal localized in a peritumoral area with marked inflammatory cell infiltration in the subcutis. (b) The circumanal glands that were localized in the dorsal resection margin area of a STS, were taking up Angiostamp™ although histologic examination was unremarkable and no $\alpha_v\beta_3$ integrin expression could be confirmed using immunohistochemistry (IHC). (c) A highly fluorescent prescapular lymph node was detected in the tumor bed after front limb amputation. The lymph node was non-metastatic and $\alpha_v\beta_3$ integrin was not expressed in the lymphatic tissue. (d) After partial resection of the scapula the resected bone *ex vivo* and the residual scapula *in vivo* showed a high NIRF-signal without evidence of tumor cell infiltration in the bone. (e) An unspecific accumulation of Angiostamp™ was intraoperatively observed in a stifle joint after tumor resection in a dog without the evidence of neoplasia or history of a stifle disease. (f) Due to the localization of a recurrent STS on the scrotum, a castration and scrotal ablation was performed. The testicle and epididymis were highly fluorescent, but STS infiltration and $\alpha_v\beta_3$ integrin expression could be ruled out by histology and IHC. (g) During metastasectomy through a partial lung lobectomy, a clear fluorescent signal was observed in the lung nodule *in vivo* intraoperatively and during imaging of the FFT *ex vivo*. Histologic examination confirmed the diagnosis of a STS metastasis.

2.4. NIRF imaging of the tumor in situ, native tissue biopsies, native tumor specimen, the tumor bed, and formalin fixed material

Table 6: Signal ratios and Dice coefficient using images of the transcutaneous tumor images and the native tumor biopsies.

Patient	Tumor in-situ (transcutaneous)								Native biopsies			
	Signal ratios							DC	Signal ratios			
	ROI T: BG	NIR T: BG	NIR T: 1cm area	1cm area: 3cm area	1cm area: BG	NIR T: 3cm area	3cm area: BG	NIR T to visibe T	Tumor: BG	1cm: BG	3cm:BG	Tumor bed: BG
1	7.8	4.4				3.7	1.6	0.6	7.2	2.3		1.0
4	4.3	4.1	2.3	2.6	1.8	2.6	1.6	0.7	22.8	3.4		1.0
6	1.9	1.7				1.4	1.2	0.5	3.1	3.3		1.6
7	14.0	10	5.0	1.4	2.0	7.1	1.4	1.0	6.8	3.4		1.3
8	5.0	3	1.9	1.1	1.5	2.1	1.4	0.9	10.3	2.4	2.5	1.9
11	2.7	2.3	1.5	1.2	1.6	1.8	1.3	0.4	2.7	2.1	1.7	1.4
12	2.5	2.2	1.4	1.7	1.6	1.7	1.3	0.7	4.2	2.5	1.6	1.3
15									5.9	4.6	1.1	1.3
16									7.1	1.8	1.4	0.9
18	1.7	1.8	1.9	0.7	1.4	2.7	0.7	0.4	4.0	2.6	1.7	1.3

BG, background; DC, Dice coefficient; Description of the ROIs in Materials and Methods of SI 4

In the transcutaneous tumor images, the fluorescent area (NIR T) and the visible tumor mass area (visibe T) were compared using the DC. The fluorescent area did not accurately reflect the area of the visible tumor mass with a mean DC of 0.65±0.22 (range: 0.4-1).

Table 7: Signal ratios and Dice coefficient using images of the native tumor specimen, the tumor bed, and formalin fixed tissue. native tumor biopsies.

Patient	Native specimen								Tumor bed		Formalin			
	Signal ratios							DC	Signal ratios		Signal ratios		DC	
	ROI T: BG	NIR T: BG	NIR T: 1cm area	1cm area: 3cm area	1cm area: BG	NIR T: 3cm area	3cm area: BG	NIR T to visibe T	Tumor bed: BG	NIR high: BG	TBR	TPR	NIR T to HE T	visible tissue to HE tissue
1	3.6	3.3				2.9	1.2		1.5		3.8	3.7	0.8	0.9
4	2.6	3	2.2	1.1	1.4	2.4	1.2	0.8	0.9	1.3	19.6	5.2	0.8	0.9
6	2.8	2.6				2.0	1.3				10.6	3.2	0.8	0.9
7	14.9	12.6	5.3	0.4	6.5	5.3	2.4	0.9	0.8	1.3	25.0	3.2	0.7	0.9
8	3.5	3	0.8	1.5	2.5	1.8	1.6	0.9	1.7	3.3	23.3	4.4	0.8	0.8
11	6.2	6.1	3.0	1.5	0.5	4.4	1.4		0.7	1.8	4.7	2.2	0.7	0.9
12	4.4	3.9	1.9	2.4	2.1	2.4	1.6	0.7			7.9	3.8	0.8	0.9
15											6.0	3.4	0.7	0.9
16											8.5	3.8	0.6	0.9
18	13.2	6.3					2.1	0.4	1.0	2.2	10.9	3.2	0.7	0.8

BG, background; DC, Dice coefficient; Description of the ROIs in Materials and Methods of SI 4

In the images of the native tumor specimen with the skin turned towards the camera head, the fluorescent area did not accurately reflect the area of the visible tumor mass with a mean DC of 0.7 (range: 0.4-0.9) (Table 7). Imaging of the specimen with the deep side turned towards the camera head revealed a TBR ≥3 in 8/8 patients (mean 6.2±3. 0). In one dog the muscle tissue on the deep side of the tumor specimen completely absorbed the NIRF-signal.

3. References

1. Dindo D, Demartines N, Clavien PA. Classification of surgical complications: a new proposal with evaluation in a cohort of 6336 patients and results of a survey. Ann Surg. 2004;240:205-13. doi:10.1097/01.sla.0000133083.54934.ae.
2. Wenk CH, Ponce F, Guillermet S, Tenaud C, Boturyn D, Dumy P, et al. Near-infrared optical guided surgery of highly infiltrative fibrosarcomas in cats using an anti-αvß3 integrin molecular probe. Cancer Lett. 2013;334:188-95. doi:10.1016/j.canlet.2012.10.041.
3. Mery E, Golzio M, Guillermet S, Lanore D, Le Naour A, Thibault B, et al. Fluorescence-guided surgery for cancer patients: a proof of concept study on human xenografts in mice and spontaneous tumors in pets. Oncotarget. 2017;8:109559-74. doi:10.18632/oncotarget.22728.
4. Chiti LE, Ferrari R, Roccabianca P, Boracchi P, Godizzi F, Busca GA, et al. Surgical Margins in Canine Cutaneous Soft-Tissue Sarcomas: A Dichotomous Classification System Does Not Accurately Predict the Risk of Local Recurrence. Animals (Basel). 2021;11. doi:10.3390/ani11082367.
5. Holt D, Parthasarathy AB, Okusanya O, Keating J, Venegas O, Deshpande C, et al. Intraoperative near-infrared fluorescence imaging and spectroscopy identifies residual tumor cells in wounds. J Biomed Opt. 2015;20:76002. doi:10.1117/1.JBO.20.7.076002.
6. Graf R, Pospischil A, Guscetti F, Meier D, Welle M, Dettwiler M. Cutaneous Tumors in Swiss Dogs: Retrospective Data From the Swiss Canine Cancer Registry, 2008-2013. Vet Pathol. 2018;55:809-20. doi:10.1177/0300985818789466.
7. Sbaraglia M, Bellan E, Dei Tos AP. The 2020 WHO Classification of Soft Tissue Tumours: news and perspectives. Pathologica. 2021;113:70-84. doi:10.32074/1591-951X-213.
8. Thway K, Noujaim J, Jones RL, Fisher C. Dermatofibrosarcoma protuberans: pathology, genetics, and potential therapeutic strategies. Ann Diagn Pathol. 2016;25:64-71. doi:10.1016/j.anndiagpath.2016.09.013.
9. McCollum KJ, Jour G, Al-Rohil RN. Cutaneous inflammatory myofibroblastic tumor with CARS-ALK fusion: Case report and literature review. J Cutan Pathol. 2022;49:795-801. doi:10.1111/cup.14261.
10. Siemion K, Reszec-Gielazyn J, Kisluk J, Roszkowiak L, Zak J, Korzynska A. What do we know about inflammatory myofibroblastic tumors? - A systematic review. Adv Med Sci. 2022;67:129-38. doi:10.1016/j.advms.2022.02.002.
11. Houdek MT, Tsoi KM, Mallett KE, Claxton RM, Ferguson PC, Griffin AM, et al. Surgical Outcomes of Primary Dermatofibrosarcoma Protuberans: A Retrospective, Multicenter Study. Ann Surg Oncol. 2022;29:8632-8. doi:10.1245/s10434-022-12351-0.
12. Lowe GC, Onajin O, Baum CL, Otley CC, Arpey CJ, Roenigk RK, et al. A Comparison of Mohs Micrographic Surgery and Wide Local Excision for Treatment of Dermatofibrosarcoma Protuberans

With Long-Term Follow-up: The Mayo Clinic Experience. Dermatol Surg. 2017;43:98-106. doi:10.1097/DSS.0000000000000910.

13. Sultan I, Qaddoumi I, Yaser S, Rodriguez-Galindo C, Ferrari A. Comparing adult and pediatric rhabdomyosarcoma in the surveillance, epidemiology and end results program, 1973 to 2005: an analysis of 2,600 patients. J Clin Oncol. 2009;27:3391-7. doi:10.1200/JCO.2008.19.7483.
14. Reifs CM, Salido-Vallejo R. Dermatofibrosarcoma protuberans with fibrosarcomatous transformation. An Bras Dermatol. 2016;91:700-1. doi:10.1590/abd1806-4841.20164886.
15. Ossendorf C, Studer GM, Bode B, Fuchs B. Sclerosing epithelioid fibrosarcoma: case presentation and a systematic review. Clin Orthop Relat Res. 2008;466:1485-91. doi:10.1007/s11999-008-0205-8.
16. Vodanovich DA, Spelman T, May D, Slavin J, Choong PFM. Predicting the prognosis of undifferentiated pleomorphic soft tissue sarcoma: a 20-year experience of 266 cases. ANZ J Surg. 2019;89:1045-50. doi:10.1111/ans.15348.
